# Mechanical Circulatory Support During Ventricular Tachycardia Ablation: A Systematic Review and Meta‐Analysis of Procedural and Clinical Outcomes

**DOI:** 10.1111/jce.70373

**Published:** 2026-05-18

**Authors:** Ahmed Nazmy, Hassan El‐Shirbiny, Alaa Abdrabou Abouelmagd, Noha Hammad, Samar Mohamed Sadek, Ahmed Wahdan Kasem, Ahmed Elazab, Reda Biomy, Islam Saboukh, Mohamed Kamal salama, Mohamed Elsaid Abdelfatah, Wael Anwar Haseeb, Maxim Didenko, Vanessa Sciacca, Thomas Fink, Angeliki Darma, Denise Guckel, Moneeb Khalaph, Christian Sohns, Philipp Sommer

**Affiliations:** ^1^ Faculty of Medicine Kafr‐Elsheikh University Kafr‐Elsheikh Egypt; ^2^ Department of Electrophysiology, Heart and Diabetes Center NRW Ruhr University Bochum Bad Oeynhausen Germany; ^3^ Cardiology Department, Faculty of Medicine Kafr Elsheikh University Kafr El Sheikh Egypt; ^4^ Faculty of Medicine South Valley University Qena Egypt; ^5^ Faculty of Medicine Port‐Said University Port‐Said Egypt; ^6^ Department of Cardiology, Faculty of Medicine Assiut University Assiut Egypt; ^7^ Faculty of Medicine Al‐Azhar University Cairo Egypt

**Keywords:** heamodynamic instability, meta‐analysis, systematic review, temporary mechanical circulatory support, ventricular tachycardia

## Abstract

Catheter ablation is an established therapy for ventricular tachycardia (VT); however, hemodynamic instability frequently limits procedural mapping and success. Temporary mechanical circulatory support (tMCS) devices are increasingly used to maintain end‐organ perfusion during VT ablation, yet their impact on clinical outcomes remains uncertain. We aimed to evaluate the impact of tMCS on clinical outcomes and periprocedural complications in patients undergoing VT ablation.

A systematic search of PubMed/MEDLINE, Scopus, Web of Science, and Cochrane CENTRAL was conducted from database inception through December 2025. We included observational studies enrolling adult patients undergoing VT ablation with tMCS, compared with VT ablation performed without MCS. Primary outcomes were all‐cause mortality, in‐hospital mortality, procedural success, VT recurrence, and major adverse cardiovascular events (MACE).

Ten observational studies comprising 16,838 patients were included, of whom 1402 received tMCS. There was no significant difference in all‐cause mortality between tMCS and no‐MCS groups (RR 1.71, 95% CI 0.68 to 4.26), procedural success (RR 1.05, 95% CI 0.92 to 1.21), VT recurrence (RR 0.99, 95% CI 0.80 to 1.22), or MACE (RR = 0.79, 95% CI: 0.62 to 1.01). However, tMCS use was associated with significantly higher in‐hospital mortality (RR 7.41, 95% CI 4.77 to 11.66). The tMCS group also demonstrated increased risks of stroke, pericardial effusion or tamponade, and periprocedural complications.

In non‐randomized studies, tMCS during VT ablation was not associated with improved long‐term outcomes, while higher in‐hospital mortality and complications likely reflected patient selection and timing of support rather than adverse device effects.

## Graphical abstract

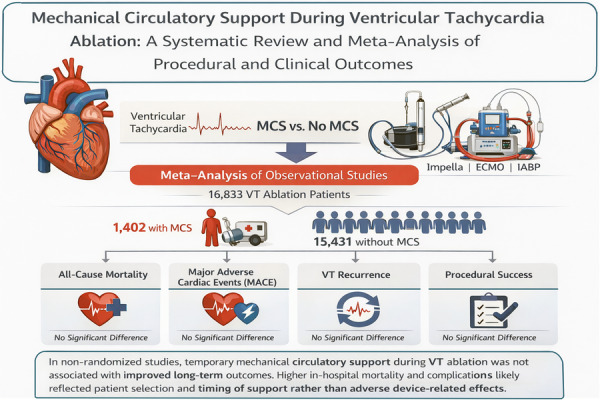

## 1 Introduction

Ventricular tachycardia (VT) is a life‐threatening arrhythmia that often occurs in patients with structural heart disease (SHD), especially those with ischemic or non‐ischemic cardiomyopathy. The management of scar‐related VT has evolved substantially over recent decades, with catheter ablation emerging as an effective therapeutic strategy to decrease to reduce recurrent VT episodes and the burden of implantable cardioverter‐defibrillator (ICD) shocks [1]. However, VT ablation procedures are often complicated by hemodynamic instability, as approximately 50%–90% of VTs are poorly tolerated, limiting the detailed activation and entrainment mapping, which in turn would otherwise potentially guide more precise ablation strategies [2].

Inadequate end‐organ perfusion during VT ablation is multifactorial, arising from underlying structural cardiac disease, hypotension during induced or spontaneous VT episodes, volume overload related to irrigated catheter systems, and the hemodynamic effects of general anesthesia [3]. When acute hemodynamic decompensation occurs during these procedures, it carries devastating consequences, with significantly elevated postprocedural mortality rates [4, 5].

To deal with these challenges, tMCS devices are increasingly used during VT ablation procedures. Several support modalities are available, including intra‐aortic balloon pumps (IABP), percutaneous left ventricular assist devices (PLVADs) such as the Impella™ and TandemHeart™, and extracorporeal membrane oxygenation (ECMO) [1, 3]. These devices help maintain end‐organ perfusion during hemodynamically unstable VT episodes, allowing for longer mapping times and more comprehensive, precise ablation [2, 5].

Despite their potential advantages and increased clinical use, the role of tMCS in VT ablation is still debated and challenging. Many studies have shown conflicting results regarding procedure outcomes, complication rates, and long‐term mortality benefits [6]. Some evidence suggests that prophylactic tMCS use in high‐risk patients may prevent acute hemodynamic collapse [5, 6], whereas other studies have found no significant improvement in outcomes despite increased procedural complexity and costs [6, 7].

Given these uncertainties and their substantial impact on a vulnerable patient population, a comprehensive review and meta‐analysis of the available evidence may help guide clinical decision‐making in routine practice. This systematic review and meta‐analysis aims to clarify the real effects of tMCS on procedural success, complication rates, and both short‐ and long‐term outcomes for patients undergoing VT ablation, thereby informing evidence‐based clinical decision‐making.

## 2 Methods

This systematic review and meta‐analysis were conducted in accordance with the Preferred Reporting Items for Systematic Reviews and Meta‐Analyses (PRISMA) statement [8] and the methodological guidance outlined in the Cochrane Handbook for Systematic Reviews of Interventions [9]. The study protocol was registered in PROSPERO (ID: [**CRD420251274810**]).

### 2.1 Eligibility Criteria and Outcomes

We included observational studies evaluating clinical outcomes associated with tMCS in adult patients (≥ 18 years) undergoing catheter ablation for VT, without restrictions on follow‐up duration. The interventional group comprised of patients who received tMCS, including pLVADs (Impella™ 2.5, Impella™ CP, TandemHeart™), IABP, or ECMO applied either prophylactically or as rescue therapy for intraprocedural hemodynamic instability, whereas the control group consisted of patients undergoing VT ablation without mechanical circulatory support.

The primary outcomes were all‐cause mortality (including in‐hospital mortality), Major Adverse Cardiovascular Events (MACE), recurrence of VT, and procedural success. MACE and procedural success were defined according to the individual definitions used in each included study; study‐specific definitions are summarized in Supplementary Table S1. Secondary outcomes were prespecified and classified into several categories. Mortality‐related outcomes included cardiovascular death, while arrhythmia‐related outcomes comprised recurrent ICD therapies. Neurological and cardiovascular complications included stroke and decompensated heart failure. Procedural and vascular complications encompassed periprocedural complications, pericardial effusion or cardiac tamponade, vascular complications, and periprocedural bleeding requiring blood transfusion. Advanced heart failure outcomes were represented by LVAD and orthotopic heart transplantation. Procedural characteristics included total procedure duration and total radiofrequency (RF) ablation time. Periprocedural complications were defined according to each study's reported criteria Supplementary Table S2.

### 2.2 Literature Search

A comprehensive literature search was performed in [PubMed/MEDLINE, Scopus, Web of Science, Cochrane CENTRAL] from inception to December 2025. The strategy combined controlled vocabulary and keywords related to VT ablation and MCS, including: (“ventricular tachycardia” OR VT OR “ventricular arrhythmia*” OR “scar‐related VT”) AND (“catheter ablation” OR ablation OR “radiofrequency ablation” OR “VT ablation” OR “substrate‐based ablation” OR “activation mapping”) AND (“mechanical circulatory support” OR MCS OR “hemodynamic support” OR “circulatory support” OR “percutaneous ventricular assist device*” OR pLVAD OR “left ventricular assist device*” OR Impella OR “TandemHeart” OR “intra‐aortic balloon pump*” OR IABP OR “extracorporeal membrane oxygenation” OR ECMO). Reference lists of included articles and relevant reviews were manually screened to identify additional eligible studies. After removing duplicates using EndNote, the remaining studies were uploaded into the Rayyan screening tool for further evaluation [10].

Records were screened in a two‐stage process: title and abstract screening, then full‐text screening of potentially eligible studies based on predefined eligibility criteria. Any disagreement was solved by discussion and consulting a senior author. Two authors independently extracted the summary and baseline characteristics from the eligible studies employing a pre‐designed extraction sheet Table 1.

**Table 1:** Summary of included studies.

Study ID	Study design	Study or registry name	Center	Study time	Country	Sample size			Population type	Intervention	Control	Inclusion criteria	Primary outcome	Follow‐up (months)
						Total	MCS	NonMCS						
Aryana 2014	Retrospective cohort	Mercy General Hospital	Single center		Sacramento, USA	68	34	34	Patients with ischemic cardiomyopathy and non‐ICM	Patients who underwent mapping and ablation with pLVAD	Patients who underwent mapping and ablation without pLVAD	The study included patients with structural heart disease and scar‐mediated, hemodynamically unstable VT, defined by mean arterial pressure ≤ 50 mmHg and/or need for internal or external defibrillation.	VT recurrence	19+|−12
Bunch 2012	Retrospective cohort	The University of Virginia and the Intermountain Medical Center	Two study sites	October 2007 and January 2010	Murray, UT, USA	31	13	18	Patients with structural heart disease (LVEF ≤ 40% or arrhythmogenic right ventricular cardiomyopathy)	pVAD‐assisted VT ablation	Purely substrate‐based mapping and ablation for unstable VT	Patients with drug‐refractory, unstable VT and significant structural heart disease underwent contemporary ablation and were matched for key clinical characteristics.	survival, freedom from ICD therapies for the clinical VT, freedom from ICD therapies for any VT, and ICD shocks for VT or VF.	3
Grimaldi 2021	Retrospective cohort	Miulli Hospital	Single center	January 2015 and December 2019.	Bari, Italy	62	31	31	All patients had ES and hemodynamically unstable VAs symptomatic for syncope or presyncope.	Catheter ablation with ECMO support	Catheter ablation without ECMO support	All patients had electrical storm with hemodynamically unstable ventricular arrhythmias causing syncope or presyncope, defined as ≥ 3 VT/VF episodes requiring cardioversion or defibrillation within 24 h and refractory to amiodarone.	Termination of ventricular arrhythmia and non‐inducibility of clinical VT/VF at the end of the ablation procedure	24 in ECMO and 25 in No ECMO
Hashimoto 2024	Retrospective cohort	Agency for Healthcare Research and Quality's Healthcare Cost and Utilization Project (HCUP).	Multicenter	2019 to 2020	USA	14,450	945	13500	Hemodynamically unstable ventricular tachycardia (VT)	MCS group, which includes pLVAD, ECMO, and IABP	Non‐MCS group	Patients aged 18 years and above who underwent VT ablation between 2019 and 2020 were included in this analysis.	The primary outcome was in‐hospital mortality	‐
Kawamura 2025	Retrospective cohort	Mount Sinai Hospital.	Single center	January 2010 to December 2022	New York City, USA	230	115	115	Scar‐related VT	pLVAD	NonpLVAD	Patients who underwent catheter ablation for scar‐related VT	Periprocedural major adverse events were defined as complications needing unplanned interventions that led to prolonged hospitalization or long‐term disability. In contrast, periprocedural decompensated heart failure was defined as heart failure requiring extended mechanical circulatory support or post‐procedure ventilation.	10
Kusa 2017	Retrospective cohort	Mount Sinai Medical Center	Single center		New York City, USA	76	38	38	Hemodynamically unstable scar‐related VT	pLVAD (Impella 2.5, Impella CP)	Non‐pLVAD	Patients who underwent catheter ablation for hemodynamically unstable, scar‐related VT, defined as VT requiring cardioversion, antitachycardia pacing, or causing dizziness/syncope.	Outcomes included recurrent VT, heart transplantation, and all‐cause mortality. Recurrent VT was monitored by continuous in‐hospital ECG, device interrogation, and outpatient record review, and was defined as sustained VT > 30 s or requiring cardioversion or pacing for termination.	7
Mathuria 2017	Retrospective cohort	Texas Heart Institute	Single‐center	January 2009 and October 2011	Houston, TX, USA	93	36	57	Ischemic and non‐ischemic cardiomyopathy.	A pre‐emptive pLVAD, Rescue pLVAD ‐‐‐> (ImpellaTM, TandemHeartTM)	A non‐pLVAD	Patients with LVEF ≤ 40% underwent VT ablation due to recurrent, drug‐refractory VT.	Thirty‐day mortality	3
Miller 2011	Retrospective cohort	Mount Sinai Medical Center (New York, New York).	single‐center	May 2010 to November 2010	New York, USA	23	10	13	Structural heart disease and hemodynamically unstable VT	Patients underwent catheter ablation of VT with pLVAD support (Impella 2.5, ABIOMED Inc., Danvers, Massachusetts),	Patients underwent catheter ablation of VT with no pLVAD support	VT ablation was performed in patients with structural heart disease and at least one episode of hemodynamically unstable VT (mean arterial pressure < 45 mmHg), referred for management of sustained monomorphic VT and/or recurrent ICD shocks.	Successful VT termination by RF ablation during ongoing VT	3
Muser 2018	Retrospective Case‐Control‐	Hospital of the University of Pennsylvania	Single‐center	January 1, 2009, and December 31, 2015.	Philadelphia, Pennsylvania, USA.	150	75	75	High‐risk patients with structural heart disease undergoing CA of scar‐related VT,	Patients undergoing CA of scar‐related VT in whom a prophylactic pLVAD was implanted. The Impella™ 2.5 or Impella™ CP pLVAD device (Abiomed Inc., Danvers, MA) was the only mechanical support device used prophylactically in this series	Patients undergoing CA of scar‐related VT who did not undergo prophylactic pLVAD placement	Patients who received prophylactic pLVAD at the start of VT ablation were compared with propensity score‐matched controls (PAINESD score) who underwent ablation without pLVAD, with the score accounting for COPD, age > 60, ischemic cardiomyopathy, NYHA III–IV, EF < 25%, VT storm, and diabetes.	Outcomes included periprocedural acute hemodynamic decompensation, VT noninducibility at procedure end and on post‐procedure NIPS (CL > 250 ms), and procedural complications such as pericardial tamponade, vascular injury, device fracture, embolic stroke, DVT, and phrenic nerve injury during epicardial ablation.	9 [15–27]
Turagam 2017	Retrospective cohort	IVTCC	Multicenter	2002 and 2015		1,655	105	1550	Hemodynamically unstable patients Undergoing Ventricular Tachycardia Ablation	Patients received HS with percutaneous ventricular assist devices, defined as the use of the Impella 2.5 ventricular support device, ECMO, and TandemHeart.	Patients Undergoing Ventricular Tachycardia Ablation without HS	VT ablation was performed for standard indications in all patients, such as recurrent ICD shocks or VT storm despite antiarrhythmic therapy.	In‐hospital and 1‐year all‐cause mortality	17.5

Abbreviations: AKI, acute kidney injury; CA, catheter ablation; CL, cycle length; COPD, chronic obstructive pulmonary disease; dLVAD, durable left ventricular assist device; DVT, deep venous thrombosis; ES, electrical storm; ECMO, extracorporeal membrane oxygenation; HS, hemodynamic support; IABP, intra‐aortic balloon pump; ICD, implantable cardioverter‐defibrillator; LVEF, left ventricular ejection fraction; LVAD, left ventricular assist device; MCS, mechanical circulatory support; NYHA, New York Heart Association functional class; NIPS, noninvasive programmed stimulation; pLVAD, percutaneous left ventricular assist device; RF, radiofrequency; VT, ventricular tachycardia; VAs, ventricular arrhythmias.

### 2.3 Risk of Bias Assessment

We evaluated the quality of the observational studies included in this study using the Newcastle‐Ottawa Scale (NOS) [11]. NOS assesses the quality of studies concerning three main domains: population selection, comparability, and outcome assessment. The reviewers evaluated the studies as “Good quality,” “Fair quality,” or “Poor quality,” according to NOS. In case of any conflicts between the authors, they were solved by discussion and consulting a senior author (Table 2).

**Table 2:** Baseline characteristics of participant.

Study ID		Age (y), Mean (SD)	Male, *n* (%)	Heart failure NYHA class III–IV, *n* (%)	LV ejection fraction (%), Mean (SD)		Comorbidities, *n* (%)						
	Intervention Control					Cardiac arrhythmia device, *n* (%) ICD	Hypertension	Diabetes mellitus	CKD	Prior cardiac surgery	COPD	Previous catheter ablation for VT	Antiarrhythmic therapy, *n* (%) Amiodarone
Aryana 2014	pLVAD	64 [12]	28 [82]	NA	30 [11]	22 [65]	23 [68]	16 [47]	21 [62]	NA	NA	NA	32 [94]
	Non‐pLVAD	67 [12]	32 [94]	NA	34 [9]	25 [74]	23 [68]	12 [35]	18 [53]	NA	NA	NA	31 [91]
Bunch 2012	pLVAD	59.7	12 (92.3)	NA	20	11 (84.6)	NA	4 (30.8)	NA	4 (30.8)	NA	6 (46.2)	6 (46.2)
	Non‐pLVAD	62.5	15 (83.3)	NA	25	18 (100)	NA	4 (22.2)	NA	6 (33.3)	NA	7 (38.9)	15 (83.3)
Grimaldi 2021	ECMO	66 [10]	28 [90]	24 [77]	30 [10]	NA	15 [48]	6 [13]	8 [26]	NA	15 [48]	6 [13]	27 [87]
	No ECMO	69 [8]	30 [97]	23 [74]	30 [8]	NA	23 [74]	9 [29]	6 [13]	NA	18 [58]	9 [29]	30 [97]
Hashimoto 2024	MCS	65.3	860 [91]	NA	NA	NA	38 [4]	302 [32]	85 [9]	NA	142 [14]	NA	NA
	Non‐MCS	64.4	10395 [77]	NA	NA	NA	2295 [15]	4320 [32]	945 [7]	NA	2790 [16]	NA	NA
Kawamura 2025	pLVAD	64.7 (10.5)	88 (76.5)	44 (38.3)	27.5 (8.8)	94 (81.7)	81 (70.4)	28 (24.3)	31 (27.0)	43 (37.4)	9 (7.8)	27 (23.5)	71 (61.7)
	Non‐pLVAD	64.4 (12.5)	91 (79.1)	47 (40.9)	27.4 (10.7)	99 (86.1)	83 (72.2)	34 (29.6)	35 (30.4)	46 (40.0)	12 (10.4)	30 (26.1)	69 (60.0)
Kusa 2017	pLVAD	63 [11]	34 [90]	14 [37]	28 [10]	NA	NA	9 [24]	18 [47]	15 [40]	NA	10 [26]	NA
	Non‐pLVAD	66 [12]	30 [79]	14 [37]	28 [10]	NA	NA	12 [32]	13 [34]	18 [47]	NA	10 [26]	NA
Mathuria 2017	pLVAD	66.8 (12.3)	33 (91.7)	10 (27.8)	25.3 (10.8)	36 (100)	29 (85.5)	25 (69.5)	13 [36]	NA	14 (38.9)	NA	33 (91.7)
	Non‐pLVAD	64.8 [9]	47 [82]	13 [23]	28 [5]	54 [95]	49 [86]	33 [58]	22 [39]	NA	14 [25]	NA	47 [82]
Miller 2011	pLVAD	64.1 (13.4)	10 (100)	6 [60]	26 [11]	8 [80]	5 [50]	0 (0)	6 [60]	NA	NA	NA	9 [90]
	Non‐pLVAD	66.8 (16.0)	13 (100)	3 [23]	35 [17]	12 [92]	5 [39]	3 [23]	3 [23]	NA	NA	NA	12 [92]
Muser 2018	pLVAD	65.6 [12]	73 [97]	24 [32]	27.6 [10]	NA	41 [67]	15 [18]	22 [29]	NA	15 [18]	NA	NA
	Non‐pLVAD	64.6 [19]	70 [93]	26 [35]	27.6 [12]	NA	48 [64]	15 [18]	28 [37]	NA	9 [12]	NA	NA
Turagam 2017	HS	63.6 (11.2)	90 (85.7)	23 (22.1)	25.4 (11.9)	56 (53.3)	79 (76.0)	47 (44.8)	40 (38.1)	41 (39.0)	NA	0.57 (0.96)	74 (71.2)
	No HS	61.7 (14.0)	1342 (86.6)	58 (3.9)	35.6 (12.9)	921 (59.8)	754 (55.0)	311 (20.7)	447 (29.0)	450 (30.1)	NA	0.50 (0.83)	776 (52.2)

### 2.4 Statistical Analysis and Data Synthesis

Statistical analyses were performed using STATA MP version 19. Dichotomous outcomes were pooled using the DerSimonian–Laird random‐effects model and reported as risk ratios (RR) with corresponding 95% confidence intervals (CIs), while continuous outcomes were pooled using mean differences (MD) with 95% CIs. Between‐study heterogeneity was assessed using the Chi‐square test and *I*² statistic, with a *p*‐value < 0.1 or *I*² > 50% indicating significant heterogeneity. Leave‐one‐out sensitivity analyses were conducted to evaluate the influence of individual studies on the overall effect estimates. Where applicable, Galbraith plots were used to explore potential sources of heterogeneity. Publication bias and small‐study effects were assessed visually using funnel plots when sufficient studies were available, and the trim‐and‐fill method was applied as a sensitivity analysis to estimate the potential impact of missing studies on the pooled results.

## 3 Results

### 3.1 Search Results and Study Selection

**Figure 1:**
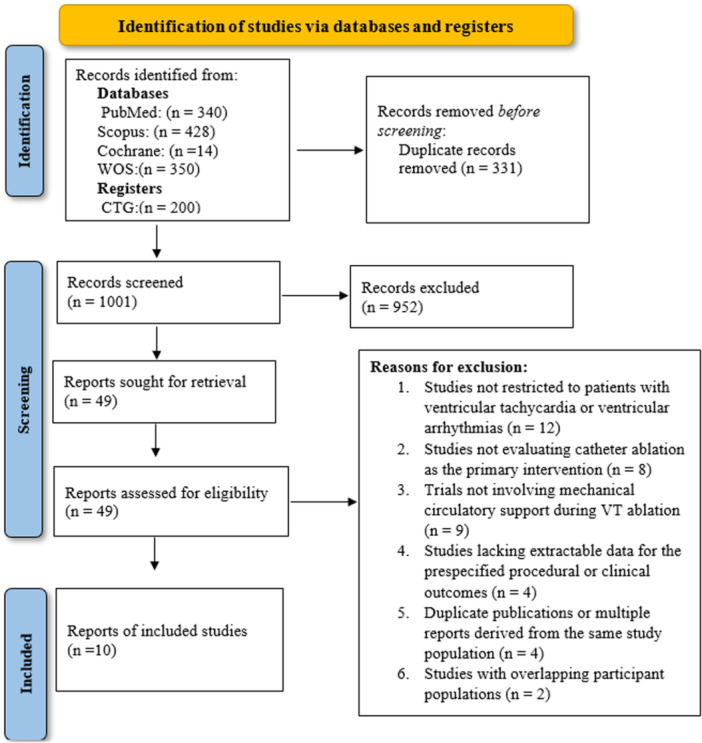
PRISMA 2020 flow diagram illustrating the study selection process.

Our initial search yielded 1332 potentially relevant articles. A total of 331 duplicate studies were deleted, and 49 studies were retained for full‐text evaluation. After excluding irrelevant studies based on our predetermined eligibility criteria, 10 eligible articles met all inclusion criteria and were included in the analysis, as shown in the PRISMA diagram (Figure 1).

### 3.2 Characteristics of Included Studies and Population

We included 10 observational studies [4, 12, 13, 14, 15, 17, 18, 19, 20, 21], totalling 16,838 patients, of whom 1402 were in the tMCS group, and 15,401 were in the no‐tMCS group. All studies were published between 2011 and 2025. When reported, the weighted mean age was 65.5 ± 11.5 years. The follow‐up duration ranges from 3 to 24 months. Detailed summary and baseline data are reported in Tables 1 and 2.

### 3.3 Risk of Bias Assessment

The quality of the included observational studies, assessed using the NOS, ranged from poor to good. Most studies were rated as good quality, with one study classified as fair (Hashimoto 2024) [19] and one as poor (Mathuria 2017) [4] (Supplementary Table S3).

### 3.4 Primary Outcomes

#### 3.4.1 All Causes of Death

**Figure 2:**
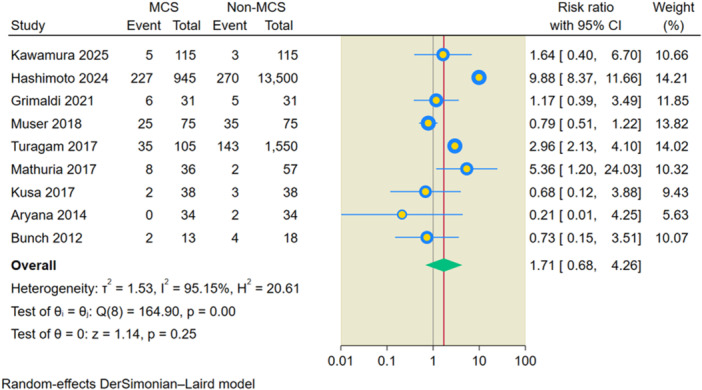
Forest plot of all‐cause mortality. ⊖₁ refers to assessments of heterogeneity between studies. ⊖ refers to the global test of differences between the treatments.

Nine studies assessed all‐cause mortality in 16,810 patients, with a pooled estimate showing no significant difference between tMCS and no‐MCS during the follow‐up period (RR = 1.71, 95% CI: 0.68 to 4.26, *p* = 0.25; *I* ^2^ = 95.15%) (Figure 2). Leave‐one‐out sensitivity analysis confirmed the robustness of these results (Supplementary Figure 1). Funnel plot analysis revealed some asymmetry around the pooled estimate (Supplementary Figure 2). The trim‐and‐fill method identified two potentially missing studies, with pooled results remaining consistent (RR = 2.17, 95% CI: 0.93–5.03) (Supplementary Figure 3).

#### 3.4.2 In Hospital Mortality

**Figure 3:**
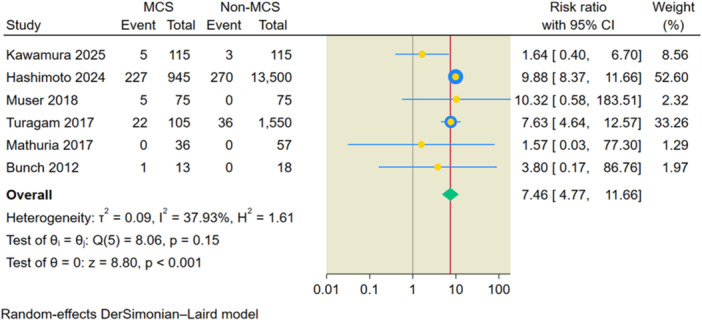
Forest plot of in‐hospital mortality. ⊖₁ refers to assessments of heterogeneity between studies. ⊖ refers to the global test of differences between the treatments.

The pooled estimate shows a higher rate of death in MCS compared to no‐MCS (RR = 7.41, 95% CI: 4.77 to 11.66, *p* < 0.001; *I* ^2^ = 37.93%) (Figure 3).

#### 3.4.3 Procedural Success

**Figure 4:**
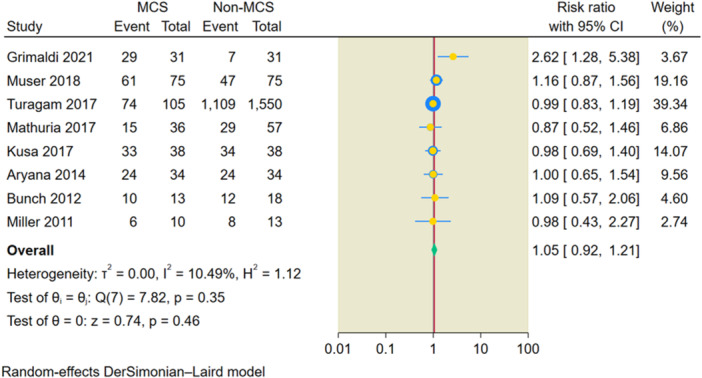
Forest plot of procedural success. ⊖₁ refers to assessments of heterogeneity between studies. ⊖ refers to the global test of differences between the treatments.

Eight studies assessed the procedural success with 2158 patients, of which the pooled estimate showed no significant difference between MCS and no‐MCS (RR = 1.05, 95% CI: 0.92 to 1.21, *p* = 0.46; *I* ^2^ = 10.49%) (Figure 4). A leave‐one‐out sensitivity analysis revealed the results remained consistent across sensitivity analyses (Supplementary Figure 4).

#### 3.4.4 VT Recurrence

**Figure 5:**
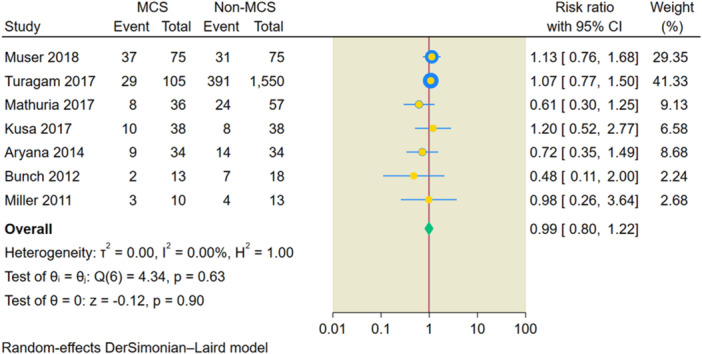
Forest plot of ventricular tachycardia recurrence. ⊖₁ refers to assessments of heterogeneity between studies. ⊖ refers to the global test of differences between the treatments.

Six studies assessed the VT recurrence with 2096 patients, of which the pooled estimate showed no significant difference between MCS and no‐MCS (RR = 0.99, 95% CI: 0.80 to 1.22, *p* = 0.90; *I* ^2^ = 0%) (Figure 5). A leave‐one‐out sensitivity analysis showed the results remained consistent across sensitivity analyses (Supplementary Figure 5).

#### 3.4.5 MACE

**Figure 6:**
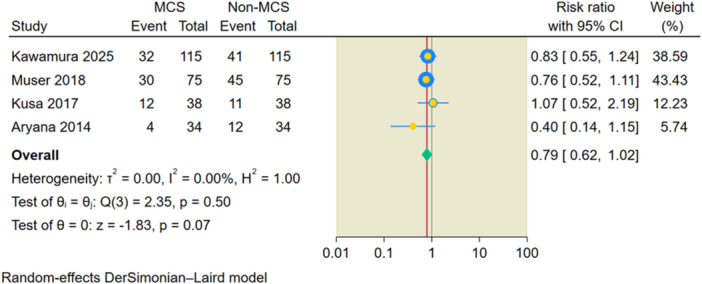
Forest plot of major adverse cardiovascular events. ⊖₁ refers to assessments of heterogeneity between studies. ⊖ refers to the global test of differences between the treatments.

Four studies assessed the incidence of MACE in 524 patients, of which the pooled estimate showed no significant difference between MCS and no‐MCS (RR = 0.79, 95% CI: 0.62 to 1.01, *p* = 0.07; I2 = 0%) (Figure 6).

#### 3.4.6 Secondary Outcomes

Clinical Outcomes. The MCS group experienced a significantly higher risk for stroke (RR = 2.85, 95% CI: 1.93 to 4.21, *p* < 001; *I* ^2^ = 0%), Pericardial Effusion or Tamponade (RR = 2.04, 95% CI: 1.07 to 3.91, *p* = 03; *I* ^2^ = 0%), Periprocedural Complications (RR = 1.88, 95% CI: 1.34 to 2.63, *p* < 001; *I* ^2^ = 0%). Conversely, we found no significant differences between the MCS and no‐MCS groups for DHF (RR = 1.19, 95% CI: 0.51 to 2.80, *p* = 0.68; *I* ^2^ = 60.57%), vascular complications (RR = 1.79, 95% CI: 0.83 to 3.85, *p* = 0.14; *I* ^2^ = 0%), CV death (RR = 1.39, 95% CI: 0.44 to 4.41 *p* = 0.57; *I* ^2^ = 42.37%), orthotopic heart transplant (RR = 0.81, 95% CI: 0.34 to 1.95 *p* = 0.64; *I* ^2^ = 0%), and epicardial ablation (RR = 1.10, 95% CI: 0.86 to 1.42 *p* = 0.45; *I* ^2^ = 0%) (Supplementary Figures 6–13).

#### 3.4.7 Procedural Outcomes

MCS group demonstrated a significantly longer fluoroscopy time (MD = 6.74, 95% CI: −4.13 to 9.35, *p* < 001; *I* ^2^ = 30.52%), procedural time (MD = 62.21, 95% CI: 32.72 to 91.67, *p* < 001; *I* ^2^ = 85.80%), and significantly less number need for VTs Induced (MD = 0.67, 95% CI: 0.47 to 0.87, *p* < 001; *I* ^2^ = 0%), However, we found no significant differences between the MCS and no‐MCS groups for inducibility of VT or VF during procedure (RR = 0.98, 95% CI: 0.65 to 1.47 *p* = 0.92; *I* ^2^ = 52.75%), inducibility of VT or VF post procedure (RR = 0.77, 95% CI: 0.35 to 1.70 *p* = 0.52; *I* ^2^ = 62.14%), and total radiofrequency ablation (MD = 2.20, 95% CI: −5.69 to 10.09, *p* = 0.58; *I* ^2^ = 81.61%) (Supplementary Figures 14–19).

## 4 Discussion

This is the most recent systematic review and meta‐analysis to comprehensively compare the outcomes of tMCS versus non‐MCS strategies for VT ablation in patients with structural heart disease. Our findings suggest that mechanical support may facilitate more comprehensive intraprocedural mapping; however, in observational studies, its use was associated with higher observed in‐hospital mortality compared with non‐supported procedures. These data emphasize the importance of patient selection when considering hemodynamic support in high‐risk VT ablation.

All‐cause mortality was a principal outcome assessed in this analysis. In pooled analyses, in‐hospital mortality was higher among patients receiving MCS, likely reflecting underlying patient acuity and selection bias rather than an adverse effect of mechanical support. This aligns with the large‐scale administrative findings of Hashimoto et al. (2024), who observed that MCS use was an independent predictor of mortality (aOR 16.4) and noted a death rate of 24% in supported patients versus 2% in controls [19]. Hashimoto et al. (2024) [19] and Turagam et al. (2019) [22] attributed this elevated mortality to confounding by indication, noting that patients selected for MCS consistently presented with higher frequencies of electrical storm, depressed ejection fractions, and advanced heart failure (NYHA class III/IV). The observed association between MCS and increased in‐hospital mortality (RR: 7.41) is a classic example of confounding by indication. In observational registries, mechanical support is preferentially reserved for rescue situation patients who have already experienced hemodynamic collapse or are in refractory electrical storm. Our results show that these patients possess a fundamentally different risk profile, with significantly lower LVEF and higher NYHA functional class [13].

To better contextualize these risks, the PAINESD score (integrating Pulmonary disease, Age, Ischemic CMP, NYHA class, EF, Storm, and Diabetes) provides a validated framework for risk stratification. When patients are compared at similar levels of baseline risk through propensity‐score matching, the mortality signal changes. For example, Muser et al. (2018) demonstrated that in high‐risk patients (median PAINESD score of 13), prophylactic pLVAD placement was actually associated with a significant reduction in the 12‐month incidence of death or heart transplantation (33% vs. 66%, *p* < 0.01). This suggests that the timing of support is a critical variable; while rescue use is associated with mortality as high as 58%, a proactive strategy based on risk scores can mitigate procedural hazards and potentially improve survival [4, 17]. The divergence between higher in‐hospital mortality and neutral long‐term mortality (RR 1.71, *p* = 0.25) suggests that, in settings where baseline risk is more evenly balanced—such as the propensity‐matched cohort reported by Kawamura et al. (2025)—mechanical support may attenuate the excess long‐term risk associated with more advanced clinical profiles, resulting in outcomes comparable to those of lower‐risk patients [14, 21].

In addition, our analysis found significantly longer procedural and fluoroscopy times in the MCS group (mean difference = 62.21 min). These metrics should not be interpreted as procedural inefficiency, but rather as indicators of expanded mapping windows and addressed substrate complexity. Mechanical support allows patients to be safely maintained in unstable VT for nearly 2.5 times longer than unsupported patients (66.7 min vs. 27.5 min, *p* = 0.03), enabling high‐fidelity activation and entrainment mapping [15]. This mapping fidelity is evidenced by the significantly higher rate of VT termination during energy delivery observed in supported procedures (90% vs. 38%) [15]. Furthermore, the stability provided by pLVADs facilitates the use of advanced, time‐consuming ablation strategies—such as bipolar ablation, needle ablation, and ethanol infusion—to target deep intramural circuits that are otherwise unaddressable in hemodynamically fragile patients [14].

It is important to recognize that the tMCS modalities included in this meta‐analysis are not physiologically equivalent. The intra‐aortic balloon pump (IABP) is a passive device that relies on rhythm synchronization; its performance is severely compromised during rapid or irregular ventricular tachycardia (VT) (>110 bpm), often leading to outcomes that mimic unsupported controls [15, 18]. In contrast, percutaneous LVADs (pLVADs) like the Impella provide active, axial flow that is independent of the native cardiac rhythm [13]. Beyond hemodynamic stabilization, pLVADs offer a distinct renal advantage; registry data indicate a significantly lower incidence of acute kidney injury (51%) compared to IABP (72%) or extracorporeal membrane oxygenation (ECMO) (70%) [19]. ECMO provides the highest level of cardiopulmonary support but increases left ventricular afterload due to retrograde flow, which may necessitate venting strategies to prevent pulmonary edema [19, 20].

Safety outcomes exhibited a trend of early favorability for the non‐MCS strategy, specifically concerning vascular access and neurological sequelae, followed by long‐term convergence. Kawamura et al. (2025) and Kusa et al. (2017) highlighted that MCS utilization resulted in significantly elevated rates of major complications and acute kidney injury, primarily driven by vascular interventions and periprocedural heart failure decompensation [14, 21]. This early hazard is likely attributable to the large‐bore arterial access necessitated by devices such as Impella and TandemHeart, alongside the physiological strain of the procedure [22]. However, concerning the pivotal variable of timing, Mathuria et al. (2017) [4] and Mariani et al. (2021) [16] identified a distinct survival advantage when support was deployed prophylactically (4% mortality) rather than as a rescue intervention (58% mortality). At the conclusion of follow‐up, pooled analyses demonstrated no significant difference in VT recurrence between supported and non‐supported ablation strategies (RR 0.99), indicating comparable long‐term arrhythmic outcomes across different procedural approaches [18, 22].

## 5 Clinical Implications

The findings support a “risk‐stratified” approach for decision‐making. For patients presenting with elevated PAINESD scores or severe baseline instability, prophylactic MCS functions as a vital stabilization mechanism that mitigates intraprocedural arrest risks, potentially enhancing long‐term survival [17]. Mathuria et al. (2017) highlighted that, while mortality was higher overall among patients receiving support, a pre‐emptive strategy demonstrated the potential to markedly reduce 30‐day mortality relative to rescue deployment (4% vs. 58%) [4]. However, this approach involves procedural risks that must be weighed against its benefits. Kawamura et al. (2025) noted that while pLVAD support enabled advanced ablation strategies and higher VT termination rates during ablation, it was associated with a cost of significantly increased periprocedural major complications and prolonged procedure times [14]. The use of mechanical support should be individualized, balancing potential procedural advantages with overall patient and procedural factors.

## 6 Limitations

This study provides a comprehensive synthesis of the available evidence on this topic, extending insights beyond those of individual small‐cohort studies. The incorporation of recent large‐scale registry data facilitates a more clinically relevant comparison between idealized outcomes and real‐world retrospective findings [13, 19]. Nevertheless, these findings should be interpreted considering several limitations.

A key limitation is the substantial heterogeneity observed in the all‐cause mortality endpoint, which may reflect differences in study populations, timing of support, and inconsistent definitions of prophylactic versus rescue support across studies. In addition, although subgroup analyses according to device type, timing of support (prophylactic vs rescue), and cardiomyopathy subtype were considered, they could not be performed in a robust manner because of the limited number of studies, heterogeneous patient populations, and inconsistent reporting across the included studies. Furthermore, the influence of selection bias in the included retrospective studies is undeniable; as highlighted by Hashimoto et al. (2024) [19] and Kawamura et al. (2025) [14], mechanical support was preferentially allocated to patients with compromised baselines (e.g., cardiogenic shock, electrical storm), potentially skewing mortality and complication metrics against the MCS group. In addition, variability in the definitions of key outcomes (procedural success, MACE, and periprocedural complications) across studies, based on individual study criteria, may have influenced pooled estimates and contributed to between‐study heterogeneity. Finally, the majority of studies reported observational data without randomization, leaving uncertainty regarding the definitive impact of these devices on long‐term survival in matched populations [16].

## 7 Conclusion

In observational studies, tMCS during VT ablation was not associated with improved long‐term outcomes compared with unsupported procedures. Higher in‐hospital mortality and complications likely reflect patient selection and baseline risk rather than device‐related effects, highlighting the importance of appropriate patient selection and timing of support. Prospective randomized studies are needed to further define the role of MCS in VT ablation.

## Funding

The authors have nothing to report.

## Conflicts of Interest

C.S. received research support and lecture fees from Medtronic, Abbott, Boston Scientific, and J&J MedTec; is a consultant for Medtronic, Boston Scientific, and J&J MedTec; has received grant support from the Else Kröner‐Fresenius‐Stiftung and Deutsche Herzstiftung. P.S. is a member of the Advisory Board for Abbott, Boston Scientific, J&J MedTech, and Medtronic. The other authors declare no conflicts of interest.

## Supporting information


**Figure S1:** Leave‐one‐out sensitivity analysis of all‐cause death.
**Figure S2:** Funnel plot of all‐cause death.
**Figure S3:** Funnel plot using the trim and fill method for All‐cause death.
**Figure S4:** leave‐one‐out sensitivity analysis of Procedural success.
**Figure S5:** leave‐one‐out sensitivity analysis of VT recurrence.
**Figure S6:** Forest plot of Stroke.
**Figure S7:** Forest plot of Pericardial Effusion or Tamponade.
**Figure S8:** Forest Plot of Periprocedural Complications.
**Figure S9:** Forest Plot of Decompensated Heart Failure.
**Figure S10:** Forest Plot of vascular complications.
**Figure S11:** Forest plot of Cardiovascular Death.
**Figure S12:** Forest plot of orthotopic heart transplant.
**Figure S13:** Forest plot of epicardial ablation.
**Figure S14:** Forest plot of total fluoroscopy time.
**Figure S15:** Forest plot of presence of procedural time.
**Figure S16:** Forest plot of VT induction number.
**Figure S17:** Forest plot of VT or VF induction during procedure.
**Figure S18:** Forest plot of VT or VF induction post procedure.
**Figure S19:** Forest plot of Total radiofrequency ablation time.
**Table S1:** Definition of MACE.
**Table S2:** Procedure Success Definitions.
**Table S3:** Periprocedural Complication Definitions.
**Table S4:** Risk of Bias assessment of observational studies by the Newcastle–Ottawa Scale (NOS).

## Data Availability

The data will be available through the supplementary appendix.

## References

[1] N. Prasitlumkum , L. Navaravong , A. Desai , et al., “Impact of Early Ventricular Tachycardia Ablation in Patients With an Implantable Cardioverter‐Defibrillator: An Updated Systematic Review and Meta‐Analysis of Randomized Controlled Trials,” Heart Rhythm: The Official Journal of the Heart Rhythm Society 19, no. 12 (2022): 2054–2061, 10.1016/j.hrthm.2022.07.005. 35820619

[2] P. Santangeli , D. Muser , E. S. Zado , et al., “Acute Hemodynamic Decompensation During Catheter Ablation of Scar‐Related Ventricular Tachycardia: Incidence, Predictors, and Impact on Mortality,” Circulation: Arrhythmia and Electrophysiology 8, no. 1 (2015): 68–75, 10.1161/CIRCEP.114.002155. 25491601

[3] P. Santangeli , D. S. Frankel , R. Tung , et al., “Early Mortality After Catheter Ablation of Ventricular Tachycardia in Patients With Structural Heart Disease,” Journal of the American College of Cardiology 69, no. 17 (2017): 2105–2115, 10.1016/j.jacc.2017.02.044. 28449770

[4] N. Mathuria , G. Wu , F. Rojas‐Delgado , et al., “Outcomes of Pre‐Emptive and Rescue Use of Percutaneous Left Ventricular Assist Device in Patients With Structural Heart Disease Undergoing Catheter Ablation of Ventricular Tachycardia,” Journal of Interventional Cardiac Electrophysiology 48, no. 1 (2017): 27–34, 10.1007/s10840-016-0168-8. 27497847

[5] Y. M. Reddy , L. Chinitz , M. Mansour , et al., “Percutaneous Left Ventricular Assist Devices in Ventricular Tachycardia Ablation: Multicenter Experience,” Circulation: Arrhythmia and Electrophysiology 7, no. 2 (2014): 244–250, 10.1161/CIRCEP.113.000548. 24532564 PMC4329420

[6] A. Aryana , A. d'Avila , C. L. Cool , et al., “Outcomes of Catheter Ablation of Ventricular Tachycardia With Mechanical Hemodynamic Support: An Analysis of the Medicare Database,” Journal of Cardiovascular Electrophysiology 28, no. 11 (2017): 1295–1302, 10.1111/jce.13312. 28800178

[7] F. Baratto , F. Pappalardo , T. Oloriz , et al., “Extracorporeal Membrane Oxygenation for Hemodynamic Support of Ventricular Tachycardia Ablation,” Circulation. Arrhythmia and electrophysiology 9 (2016): e004492, 10.1161/CIRCEP.116.004492. 27932426

[8] M. J. Page , J. E. McKenzie , P. M. Bossuyt , et al., “The PRISMA 2020 Statement: An Updated Guideline for Reporting Systematic Reviews,” BMJ 372 (2021): n71, 10.1136/bmj.n71. 33782057 PMC8005924

[9] Cochrane Handbook for Systematic Reviews of Interventions | Cochrane [Internet]. [cited 2025 Dec 29]. https://www.cochrane.org/authors/handbooks-and-manuals/handbook.

[10] M. Ouzzani , H. Hammady , Z. Fedorowicz , and A. Elmagarmid , “Rayyan—A Web and Mobile App for Systematic Reviews,” Systematic Reviews 5 (2016): 210, 10.1186/s13643-016-0384-4. 27919275 PMC5139140

[11] A. Stang , “Critical Evaluation of the Newcastle‐Ottawa Scale for the Assessment of the Quality of Nonrandomized Studies in Meta‐Analyses,” European Journal of Epidemiology 25, no. 9 (2010): 603–605, 10.1007/s10654-010-9491-z. 20652370

[12] T. J. Bunch , A. Darby , H. T. May , et al., “Efficacy and Safety of Ventricular Tachycardia Ablation With Mechanical Circulatory Support Compared With Substrate‐Based Ablation Techniques,” Europace: European Pacing, Arrhythmias, and Cardiac Electrophysiology: Journal Of The Working Groups On Cardiac Pacing, Arrhythmias, And Cardiac Cellular Electrophysiology of the European Society of Cardiology 14, no. 5 (2012): 709–714, 10.1093/europace/eur347. 22080473 PMC3598428

[13] M. K. Turagam , V. Vuddanda , D. Atkins , et al., “Hemodynamic Support in Ventricular Tachycardia Ablation: An International VT Ablation Center Collaborative Group Study,” JACC Clin Electrophysiol 3, no. 13 (2017): 1534–1543, 10.1016/j.jacep.2017.07.005. 29759835

[14] I. Kawamura , J. S. Koruth , S. Kusa , et al., “Outcomes of Scar‐Related Ventricular Tachycardia Ablation With Percutaneous Left Ventricular Assist Device Support,” JACC: Clinical Electrophysiology 11, no. 12 (2025): 2673–2684, 10.1016/j.jacep.2025.07.016. 40938229

[15] M. A. Miller , S. R. Dukkipati , A. J. Mittnacht , et al., “Activation and Entrainment Mapping of Hemodynamically Unstable Ventricular Tachycardia Using a Percutaneous Left Ventricular Assist Device,” Journal of the American College of Cardiology 58, no. 13 (2011): 1363–1371, 10.1016/j.jacc.2011.06.022. 21920266

[16] S. Mariani , L. C. Napp , K. Kraaier , et al., “Prophylactic Mechanical Circulatory Support for Protected Ventricular Tachycardia Ablation: A Meta‐Analysis of the Literature,” Artificial Organs 45, no. 9 (2021): 987–997, 10.1111/aor.13945. 33616221

[17] D. Muser , J. J. Liang , S. A. Castro , et al., “Outcomes With Prophylactic Use of Percutaneous Left Ventricular Assist Devices in High‐Risk Patients Undergoing Catheter Ablation of Scar‐Related Ventricular Tachycardia: A Propensity‐Score Matched Analysis,” Heart Rhythm: The Official Journal of the Heart Rhythm Society 15, no. 10 (2018): 1500–1506, 10.1016/j.hrthm.2018.04.028. 29753944

[18] A. Aryana , P. Gearoid O'Neill , D. Gregory , et al., “Procedural and Clinical Outcomes After Catheter Ablation of Unstable Ventricular Tachycardia Supported by a Percutaneous Left Ventricular Assist Device,” Heart Rhythm: The Official Journal of the Heart Rhythm Society 11, no. 7 (2014): 1122–1130, 10.1016/j.hrthm.2014.04.018. 24732372

[19] K. Hashimoto , A. R. Akkawi , M. Ghazal , A. Briasoulis , and T. Kuno , “Impact of Mechanical Circulatory Support on In‐Hospital Outcomes Among Patients With Ventricular Tachycardia Requiring Ablation,” Artificial Organs 49, no. 4 (2025): 681–690, 10.1111/aor.14877. 39345216

[20] M. Grimaldi , M. M. Marino , N. Vitulano , et al., “Cardiopulmonary Support During Catheter Ablation of Ventricular Arrhythmias With Hemodynamic Instability: The Role of Inducibility,” Frontiers in Cardiovascular Medicine 8 (2021): 747858, 10.3389/fcvm.2021.747858. 34746263 PMC8563579

[21] S. Kusa , M. A. Miller , W. Whang , et al., “Outcomes of Ventricular Tachycardia Ablation Using Percutaneous Left Ventricular Assist Devices,” Circulation: Arrhythmia and Electrophysiology 10, no. 6 (2017): e004717, 10.1161/CIRCEP.116.004717. 28576780

[22] M. K. Turagam , V. Vuddanda , S. Koerber , et al., “Percutaneous Ventricular Assist Device in Ventricular Tachycardia Ablation: A Systematic Review and Meta‐Analysis,” Journal of Interventional Cardiac Electrophysiology 55, no. 2 (2019): 197–205, 10.1007/s10840-018-0477-1. 30377926

