# Supplementary material for: Mechanical Circulatory Support During Ventricular Tachycardia Ablation: A Systematic Review and Meta‐Analysis of Procedural and Clinical Outcomes
Source: J Cardiovasc Electrophysiol. 2026 May 18;37(7):1564–76. doi: 10.1111/jce.70373 (PMC13372441; doi:10.1111/jce.70373)

| **Content** | **Page** |
| --- | --- |
| **Table S1:** Definition of MACE | 2 |
| **Table S2.** Procedure Success Definitions. | 3 |
| **Table S3.** Periprocedural Complication Definitions. | 4 |
| **Table S4.** Risk of Bias assessment of observational studies by the Newcastle–Ottawa Scale (NOS) | 5 |
| **Figure S1.** Leave-one-out sensitivity analysis of all-cause death. | 6 |
| **Figure S2.**  Funnel plot of all-cause death. | 7 |
| **Figure S3.** Funnel plot using the trim and fill method for All-cause death | 8 |
| **Figure S4.** leave-one-out sensitivity analysis of Procedural success. | 9 |
| **Figure S5.**  leave-one-out sensitivity analysis of VT recurrence. | 10 |
| **Figure S6.** Forest plot of Stroke. | 11 |
| **Figure S7:**  Forest plot of Pericardial Effusion or Tamponade. | 12 |
| **Figure S8:**  Forest Plot of Periprocedural Complications. | 13 |
| **Figure S9:** Forest Plot of Decompensated Heart Failure. | 14 |
| **Figure S10:** Forest Plot of vascular complications. | 15 |
| **Figure S11:** Forest plot of Cardiovascular Death. | 16 |
| **Figure S12:** Forest plot of orthotopic heart transplant. | 17 |
| **Figure S13:** Forest plot of epicardial ablation. | 18 |
| **Figure S14:** Forest plot of total fluoroscopy time. | 19 |
| **Figure S15:** Forest plot of presence of procedural time. | 20 |
| **Figure S16:** Forest plot of VT induction number. | 21 |
| **Figure S17:** Forest plot of VT or VF induction during procedure. | 22 |
| **Figure S18:** Forest plot of VT or VF induction post procedure. | 23 |
| **Figure S19:** Forest plot of Total radiofrequency ablation time. | 24 |

**Supplementary Appendix**

**Table S1:** Definition of MACE

| Study ID | Definition of MACE |
| --- | --- |
| Kawamura 2025 | Rehospitalization for VT or worsening chronic heart failure requiring hospitalization, LVAD implantation, orthotopic heart transplantation, and all-cause mortality. |
| Muser 2018 | Composite of Death/transplant |
| Kusa 2017 | Recurrent VT, heart transplant, and all-cause death |
| Aryana 2014 | 30-day rehospitalization, redo-VT ablation, recurrent ICD therapies, and 3-month mortality. |

| Table S2. Procedure Success Definitions. |
| --- |

| Study ID | Procedure Success Definition |
| --- | --- |
| Bunch et al. (2012) | "Acute procedural success, defined as the inability to induce all the VT(s) induced at the beginning of the procedure at the end of the procedure." |
| Kusa et al. (2017) | The study does not explicitly define a binary "success" variable in the methods, but measures: 1. "VT termination during ablation" and 2. "VT inducibility" at procedure conclusion. |
| Turagam et al. (2017) | "Success of VT ablation was defined as complete termination of monomorphic VT and subsequent VT noninducibility despite programmed electrical stimulation unless prohibited due to hemodynamic instability." |
| Muser et al. (2018) | "The acute procedural efficacy was assessed based on the inducibility of VT (excluding fast [<250 ms] nonclinical VTs) at the end of the ablation procedure with a consistent stimulation protocol." |
| Miller et al. (2011) | The study assesses "VT terminable by energy delivery" and "Inducibility for MMVT at conclusion of procedure." |
| Hashimoto et al. (2024) | **Not Defined**. (This is an administrative database study focusing on mortality and complications; it does not contain electrophysiological procedural success data). |
| Grimaldi et al. (2021) | "Procedural success was defined as an inability to induce sustained VTs and the disappearance of frequent spontaneous premature ventricular complexes." |
| Mathuria et al. (2017) | Not explicitly defined as "Success", but measured as "Non-inducible post-procedure." |
| Kawamura et al. (2025) | The study measures "Postprocedural VT inducibility" rather than a binary success definition. |
| Aryana et al. (2014) | "Procedural success was defined as noninducibility of any sustained monomorphic VT with programmed electrical stimulation using triple extrastimuli." |

| Table S3. Periprocedural Complication Definitions. |
| --- |

| Study ID | Periprocedural Complication Definition |
| --- | --- |
| Bunch et al. (2012) | Complications were not defined by a single composite definition but listed individually: stroke, death, pericardial effusions requiring intervention, ST segment elevation, phrenic nerve injury, or acute congestive heart failure requiring ventilation >24 h. |
| Kusa et al. (2017) | Complications monitored included: "pericardial effusion, vascular complications, and worsening heart failure." **Acute Kidney Injury (AKI)** was specifically defined as "an absolute increase in serum creatinine elevation of ≥0.3 mg/dL or increase of ≥150% within 48 hours after the procedure." |
| Turagam et al. (2017) | "Major complications such as pericardial tamponade or effusion requiring drainage as well as vascular complications requiring intervention, stroke, and intraprocedural death were recorded." |
| Muser et al. (2018) | "We defined as major complication any adverse event related to the procedure that required an unplanned intervention including invasive procedures (eg, vascular surgery, pericardiocentesis, and thrombin injection) or blood transfusions, causing long-term disability or resulting in prolonged hospitalization." |
| Miller et al. (2011) | "Major complications" were reported as specific events: "pericardial tamponade," "vascular access site injuries," "groin hematomas," "stroke/systemic embolism," "avulsion of the aortic valve." |
| Hashimoto et al. (2024) | Defined by ICD-10 codes for: "acute kidney injury (AKI), AKI-requiring dialysis, hemorrhagic stroke, gastrointestinal bleeding, any bleeding events, an eventual heart transplant, and durable LVAD (dLVAD) during hospitalization." |
| Grimaldi et al. (2021) | Not explicitly defined in methods, but reported as "procedural complications" in results (e.g., femoral artery damage, pseudoaneurysms, dissections). |
| Mathuria et al. (2017) | **Hemodynamic collapse** is defined as: "pulseless electrical activity, refractory ventricular fibrillation/tachycardia, persistent hypotension (mean arterial pressure, MAP <50 mmHg) despite vasopressor(s), and/or acute pulmonary edema." |
| Kawamura et al. (2025) | "Primary safety endpoint was defined as periprocedural major adverse events... classified as any adverse events related to the procedure that necessitated unplanned interventions... and resulted in long-term disability or prolonged hospitalization." |
| Aryana et al. (2014) | Not explicitly defined as a composite in methods. Results report "major complications" including femoral arterial laceration requiring surgery and cardiac tamponade requiring pericardiocentesis. |

**Table S4.** Risk of Bias assessment of observational studies by the Newcastle–Ottawa Scale (NOS).

| Study ID | Desgin | Selection | Comparability | Outcome | Total Stars | Quality Rating |
| --- | --- | --- | --- | --- | --- | --- |
| Bunch 2012 | Retrospective cohort | **★★★★** | **★★** | **★★** | **8/9** | **Good** |
| Grimaldi 2021 | Retrospective cohort | **★★★** | **★** | **★★** | **6/9** | **Good** |
| Hashimoto 2024 | Retrospective cohort | **★★★** | **★★** | **★** | **6/9** | **Fair** |
| Kawamura 2025 | Retrospective cohort | **★★★★** | **★★** | **★★★** | **9/9** | **Good** |
| Kusa 2017 | Retrospective cohort | **★★★★** | **★★** | **★★** | **8/9** | **Good** |
| Mathuria 2017 | Retrospective cohort | **★★★** | **★** | **★** | **5/9** | **Poor** |
| Miller 2011 | Retrospective cohort | **★★★** | **★** | **★★** | **6/9** | **Good** |
| Muser 2018 | Retrospective Case-Control- | **★★★★** | **★★** | **★★★** | **9/9** | **Good** |
| Turagam 2017 | Retrospective cohort | **★★★★** | **★★** | **★★** | **8/9** | **Good** |
| Aryana 2014 | Retrospective cohort | **★★★** | **★★** | **★★** | **7/9** | **Good** |

**Figure S1:** Leave-one-out sensitivity analysis of all-cause death.


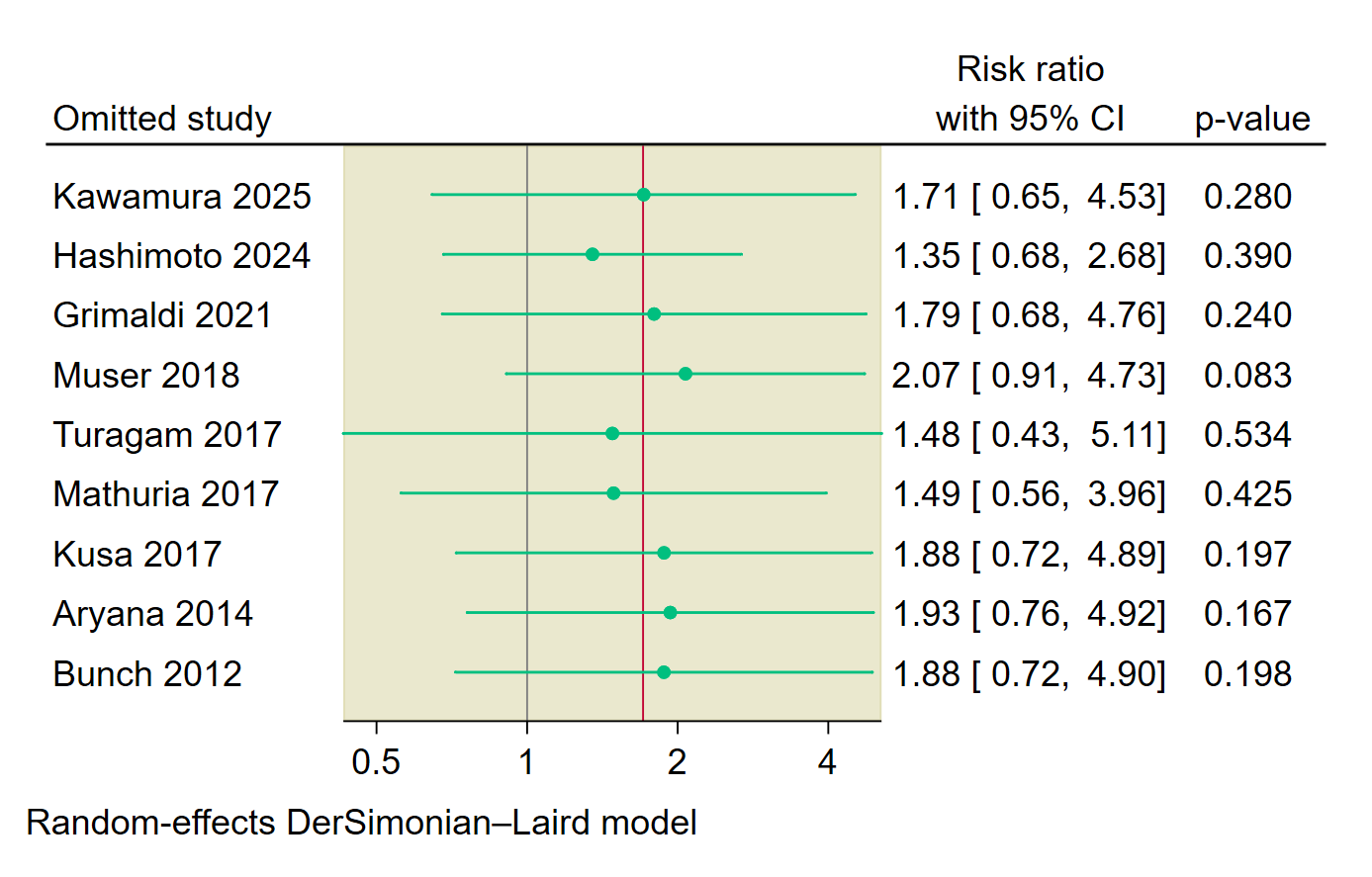


**Figure S2:** Funnel plot of all-cause death.


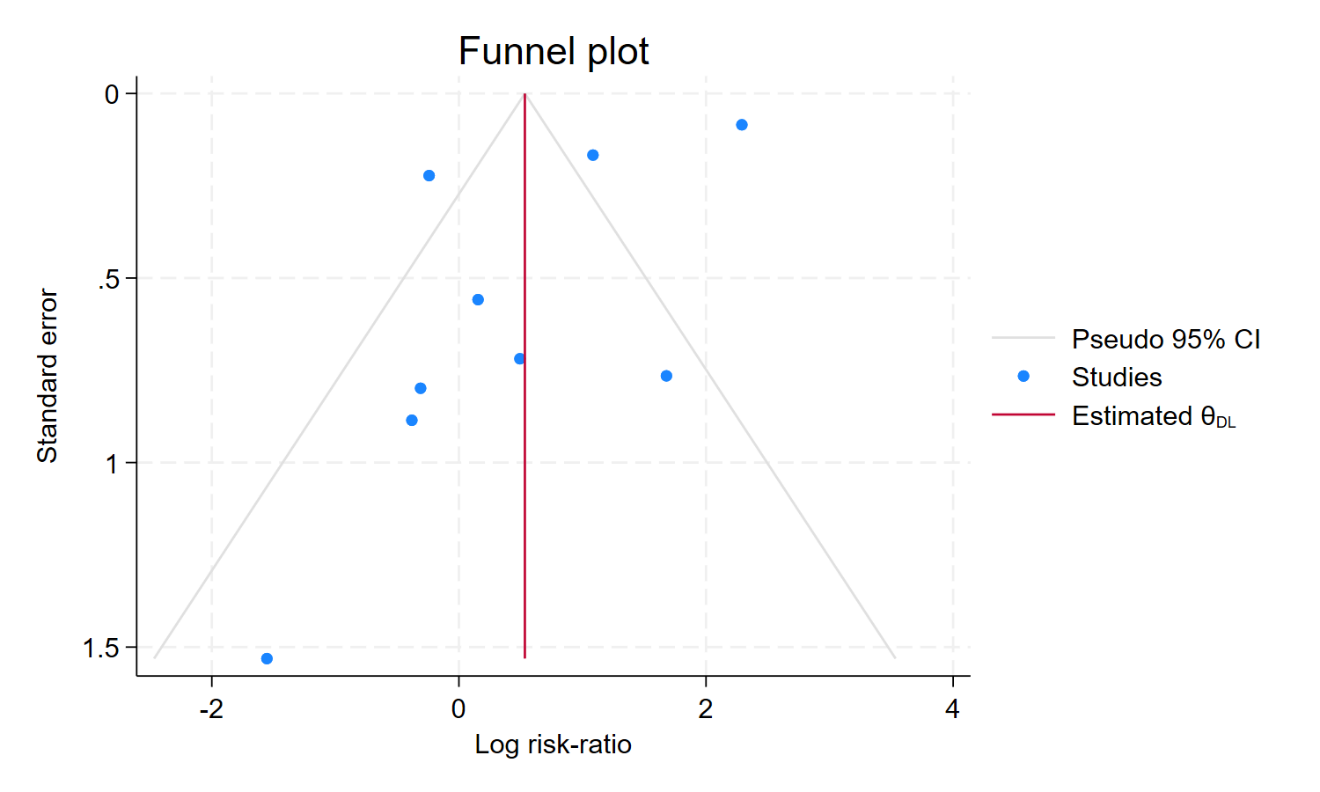


**Figure S3:** Funnel plot using the trim and fill method for All-cause death.


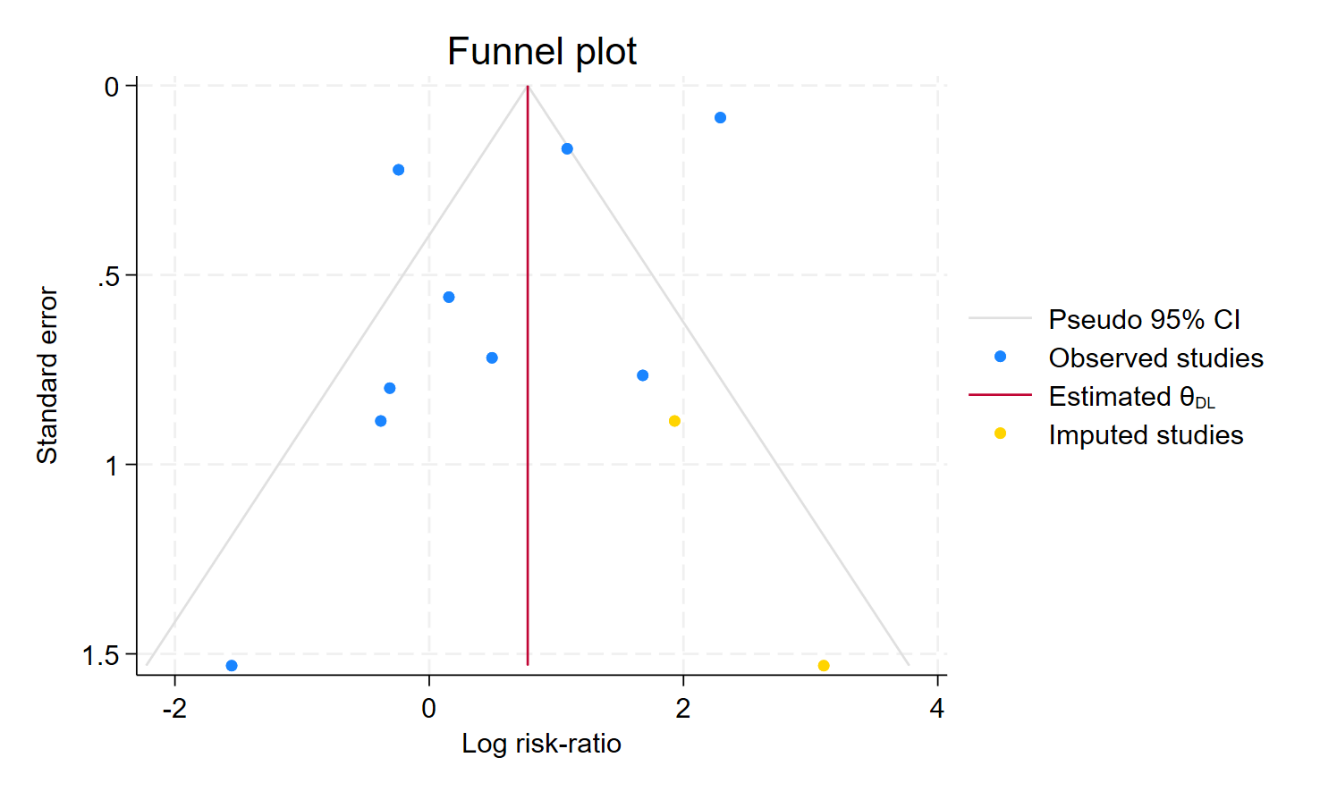


**Figure S4:** leave-one-out sensitivity analysis of Procedural success.


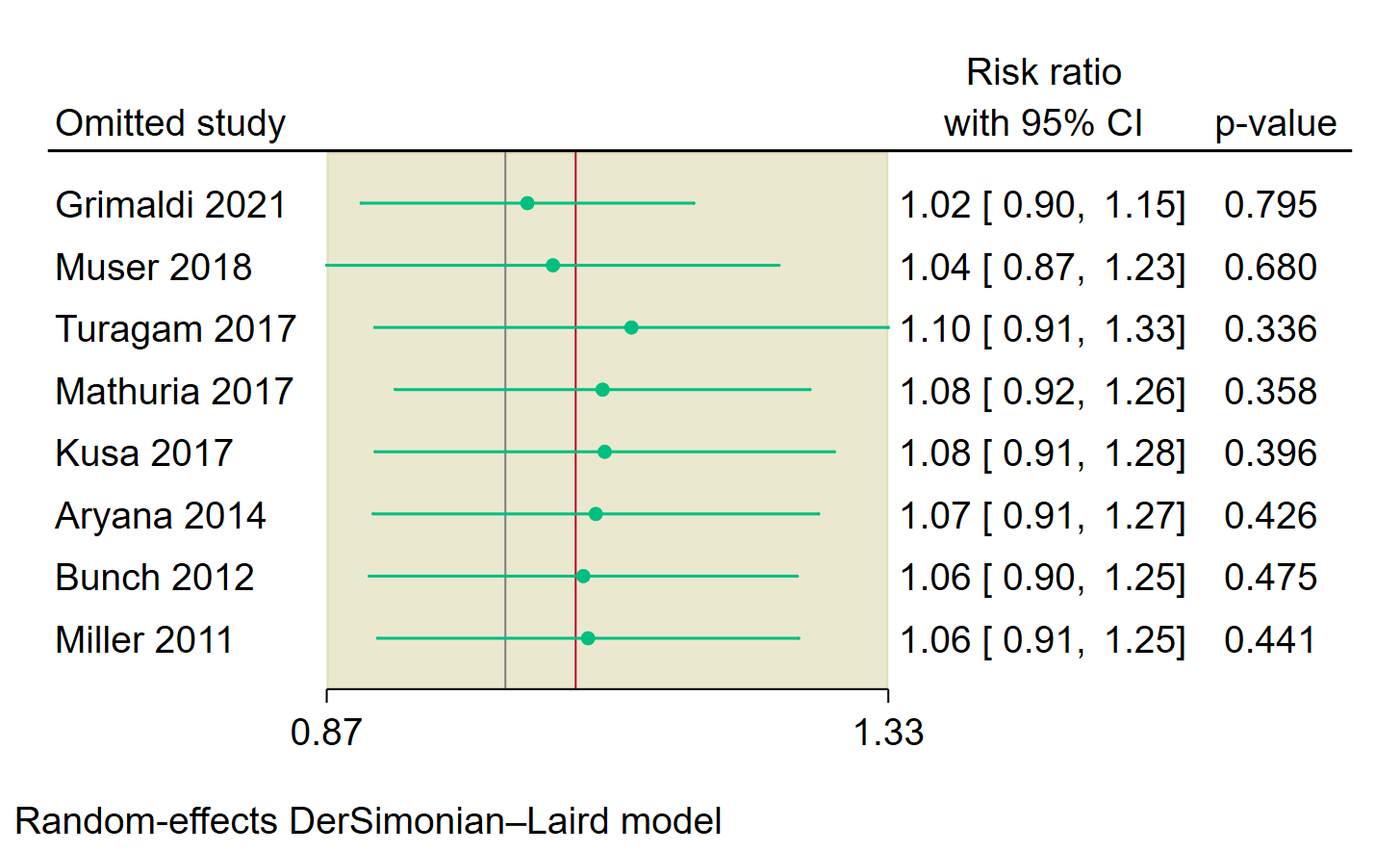


**Figure S5**: leave-one-out sensitivity analysis of VT recurrence.


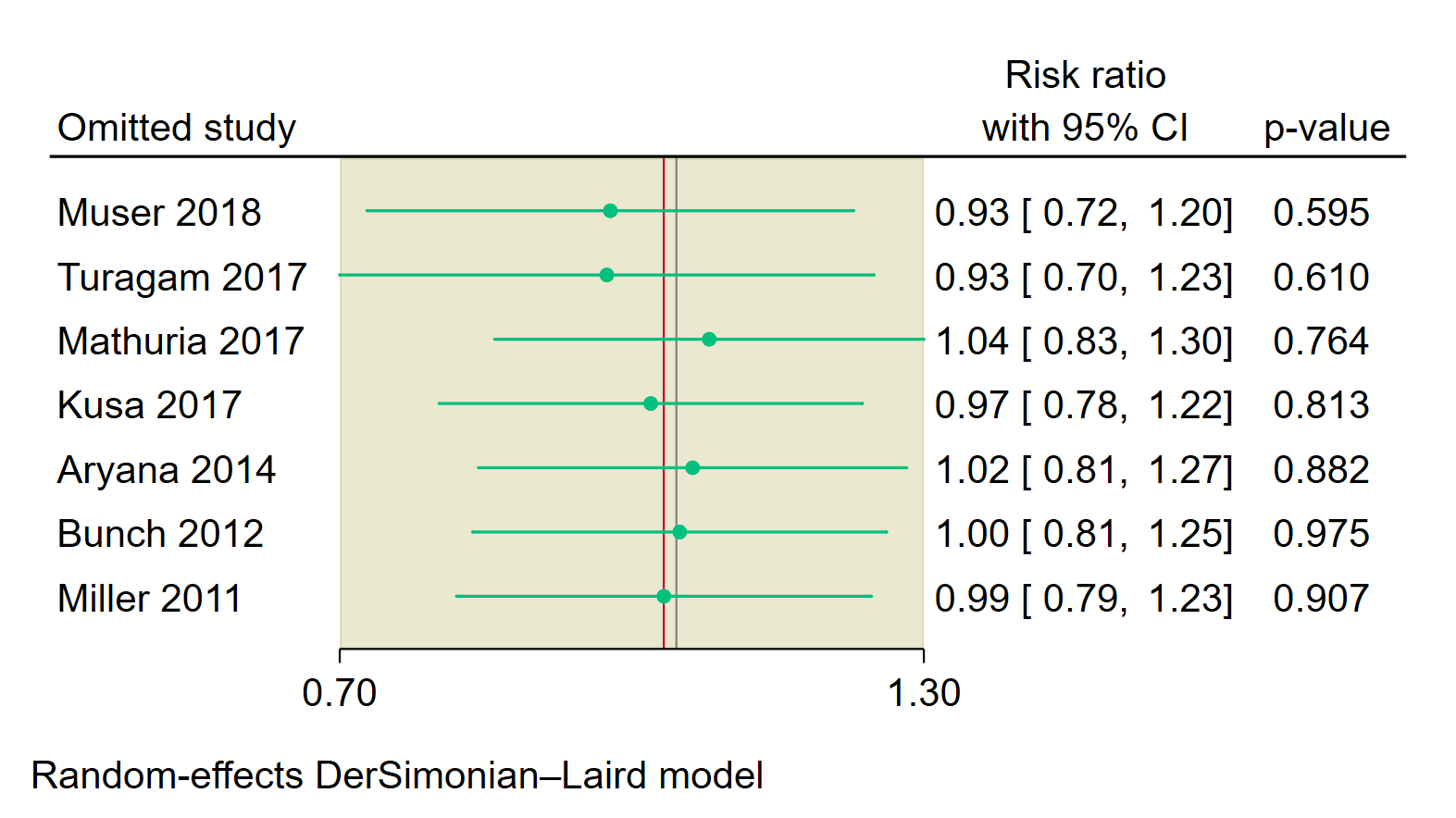


**Figure S6:** Forest plot of Stroke.

Ɵ_1_ refers to assessments of heterogeneity between studies. Ɵ refers to the global test of differences between the treatments.


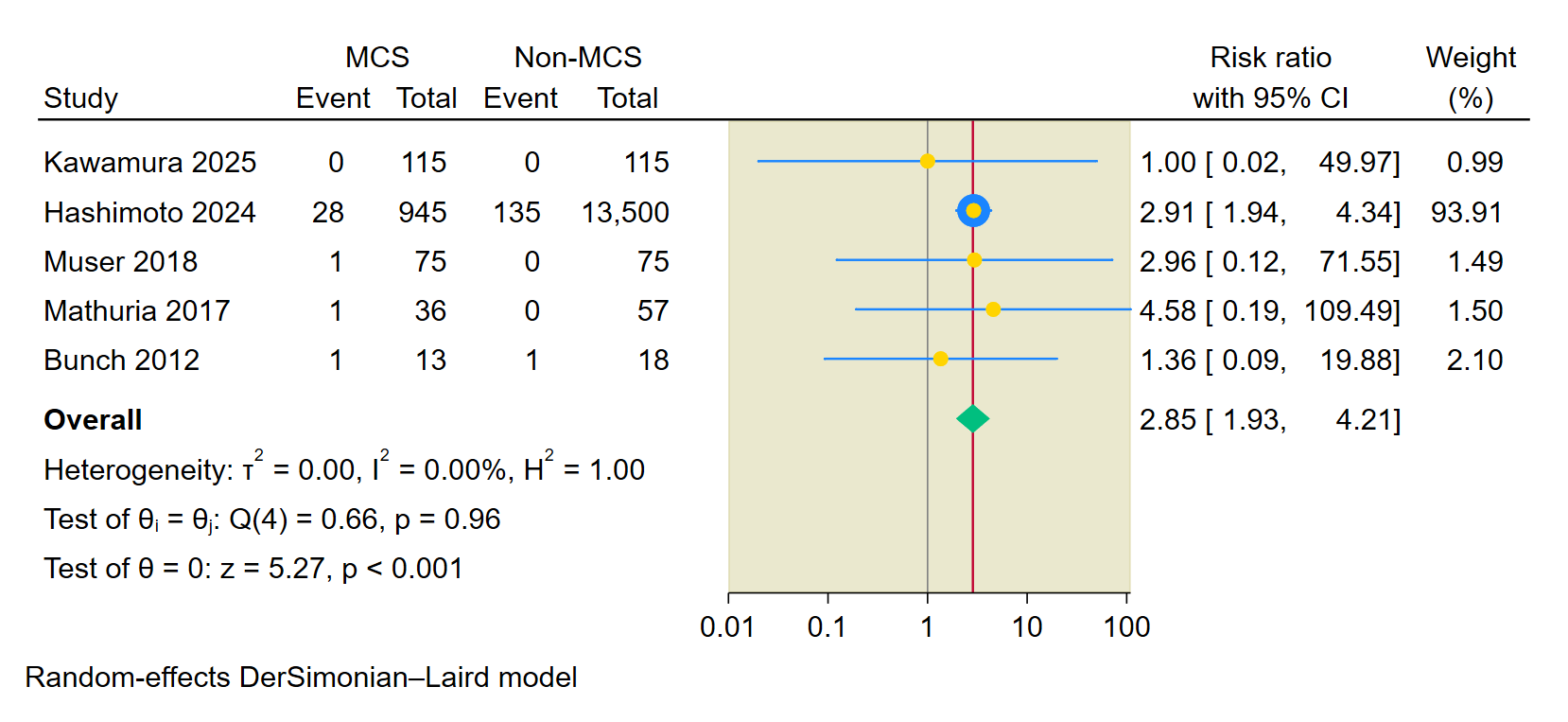


**Figure S7.** Forest plot of Pericardial Effusion or Tamponade.

Ɵ_1_ refers to assessments of heterogeneity between studies. Ɵ refers to the global test of differences between the treatments.


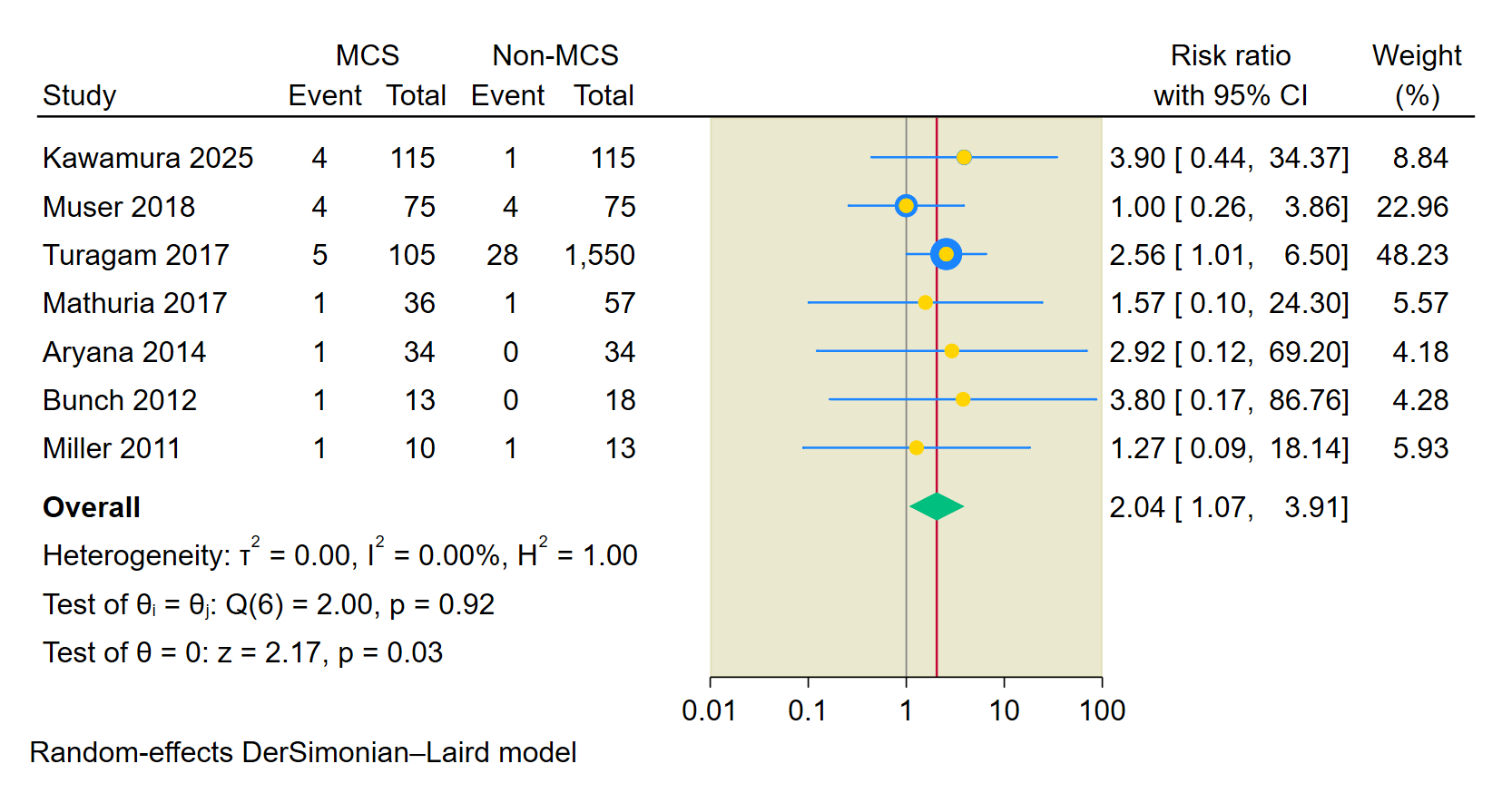


**Figure S8:** Forest Plot of Periprocedural Complications.

Ɵ_1_ refers to assessments of heterogeneity between studies. Ɵ refers to the global test of differences between the treatments.


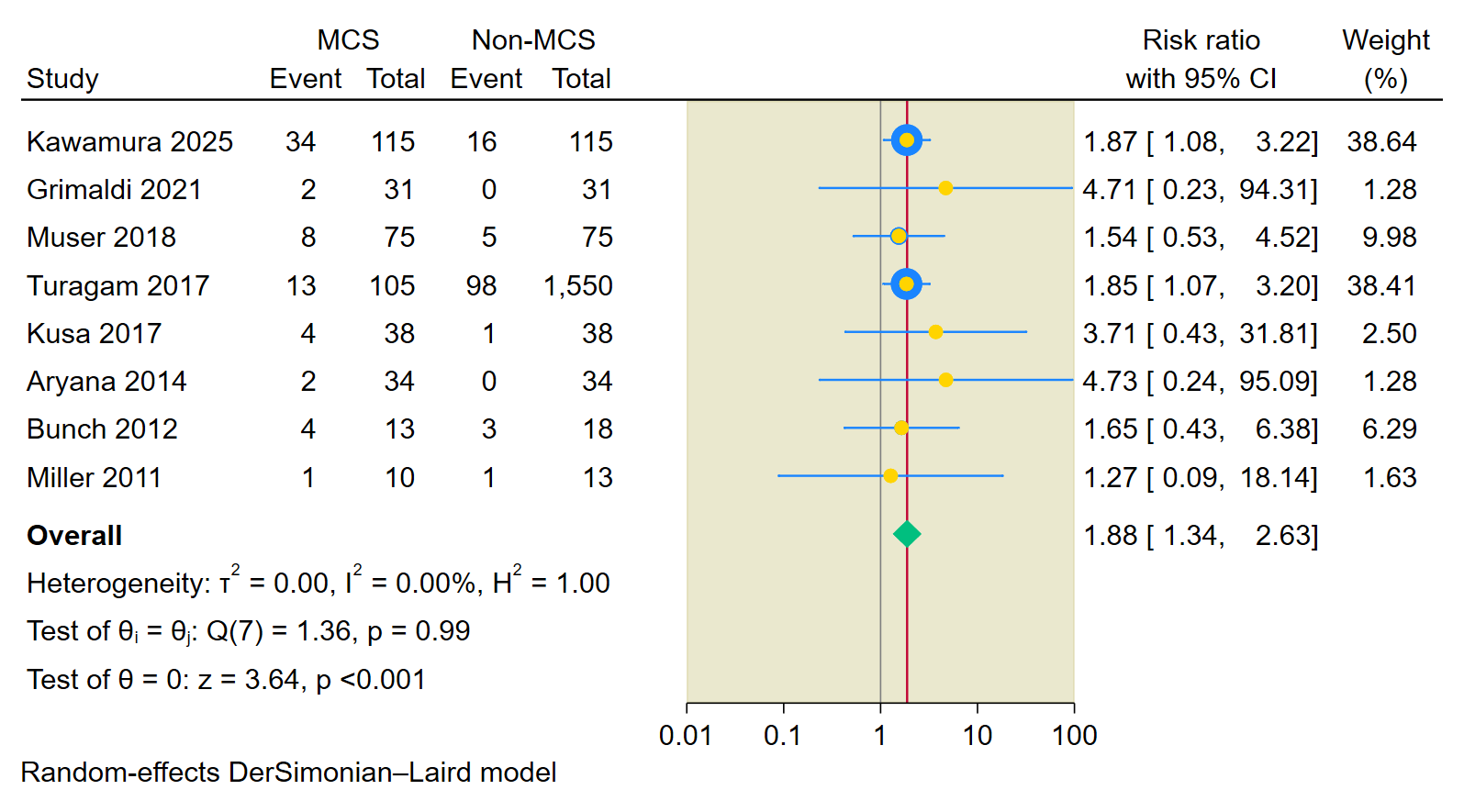


**Figure S9:** Forest Plot of Decompensated Heart Failure.

Ɵ_1_ refers to assessments of heterogeneity between studies. Ɵ refers to the global test of differences between the treatments.


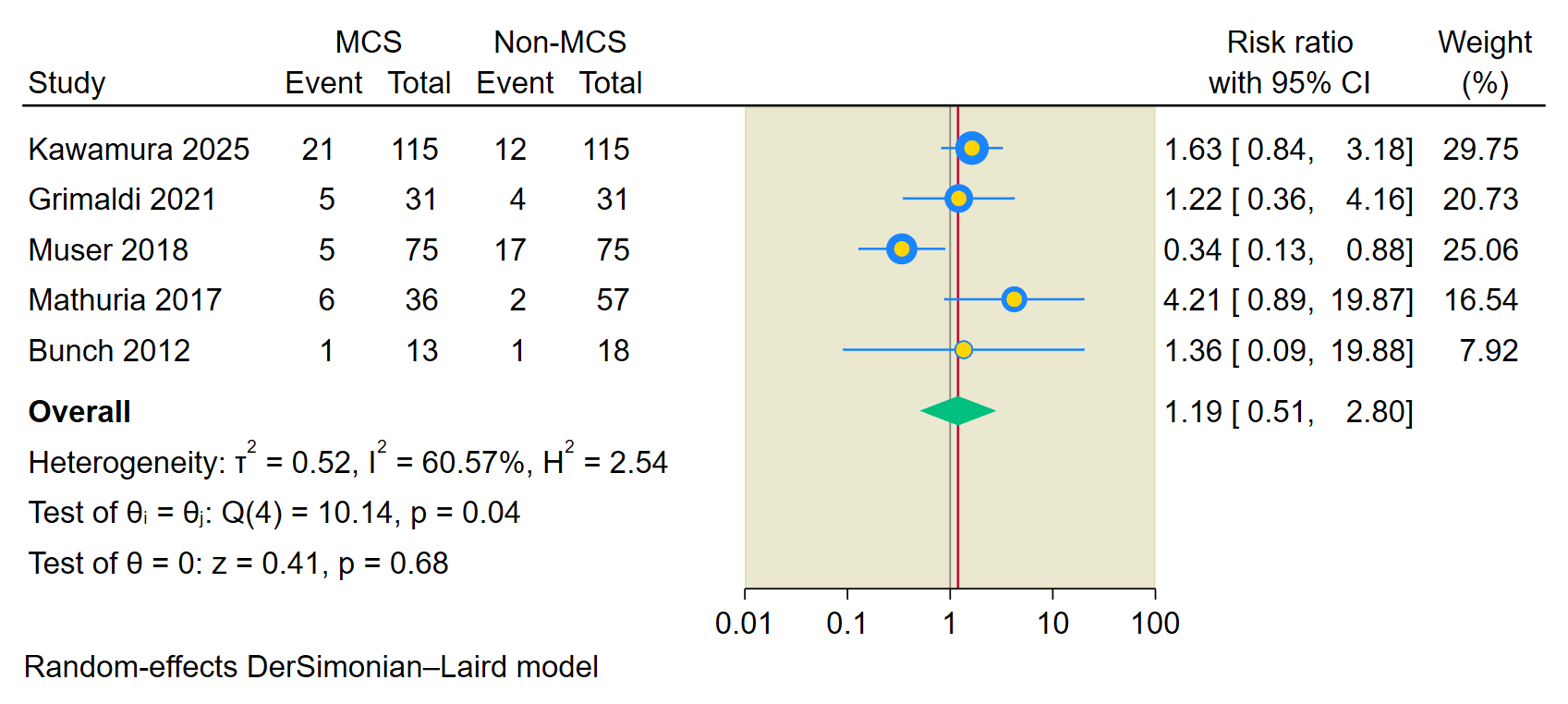


**Figure S10:** Forest Plot of vascular complications.

Ɵ_1_ refers to assessments of heterogeneity between studies. Ɵ refers to the global test of differences between the treatments.


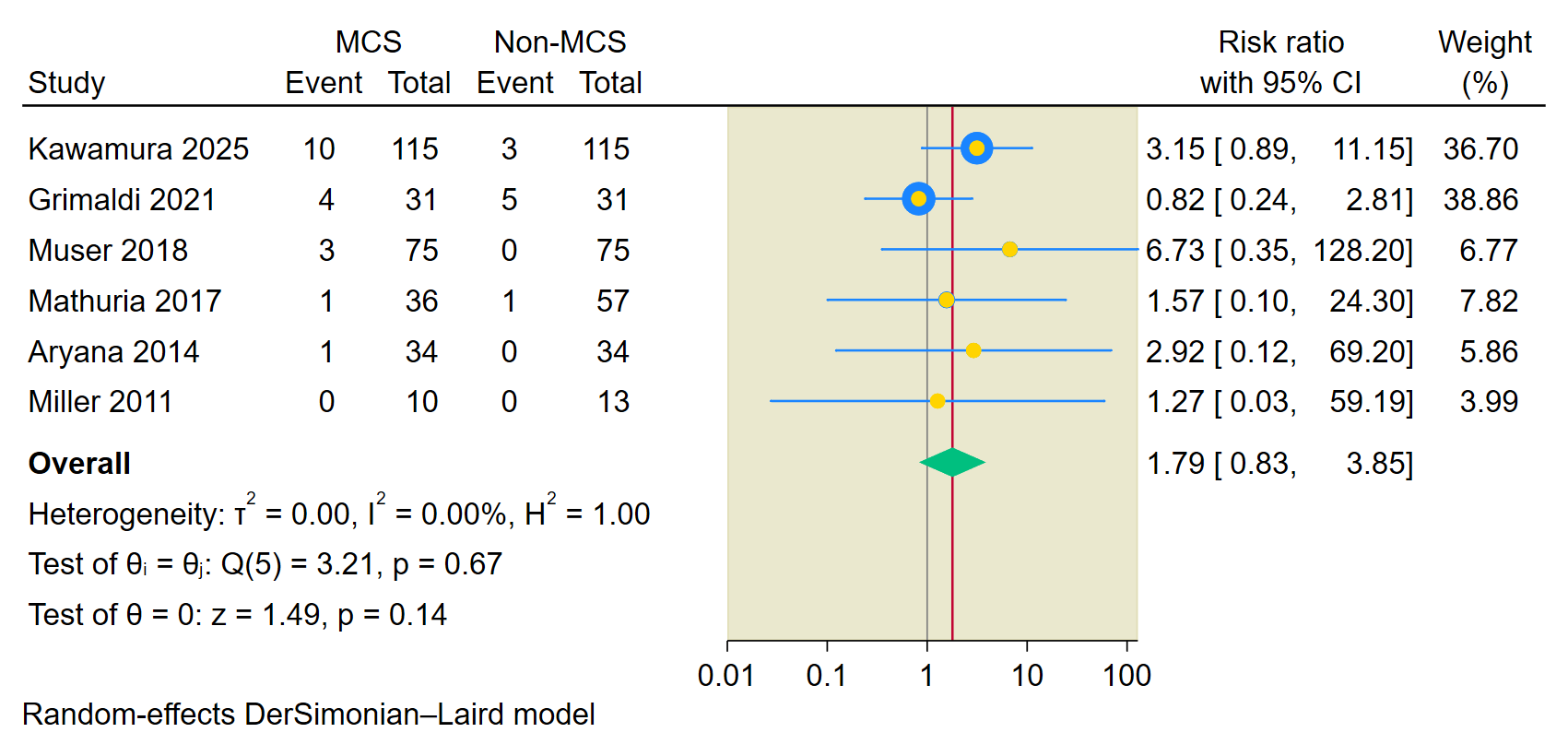


**Figure S11:** Forest plot of Cardiovascular Death.

Ɵ_1_ refers to assessments of heterogeneity between studies. Ɵ refers to the global test of differences between the treatments.


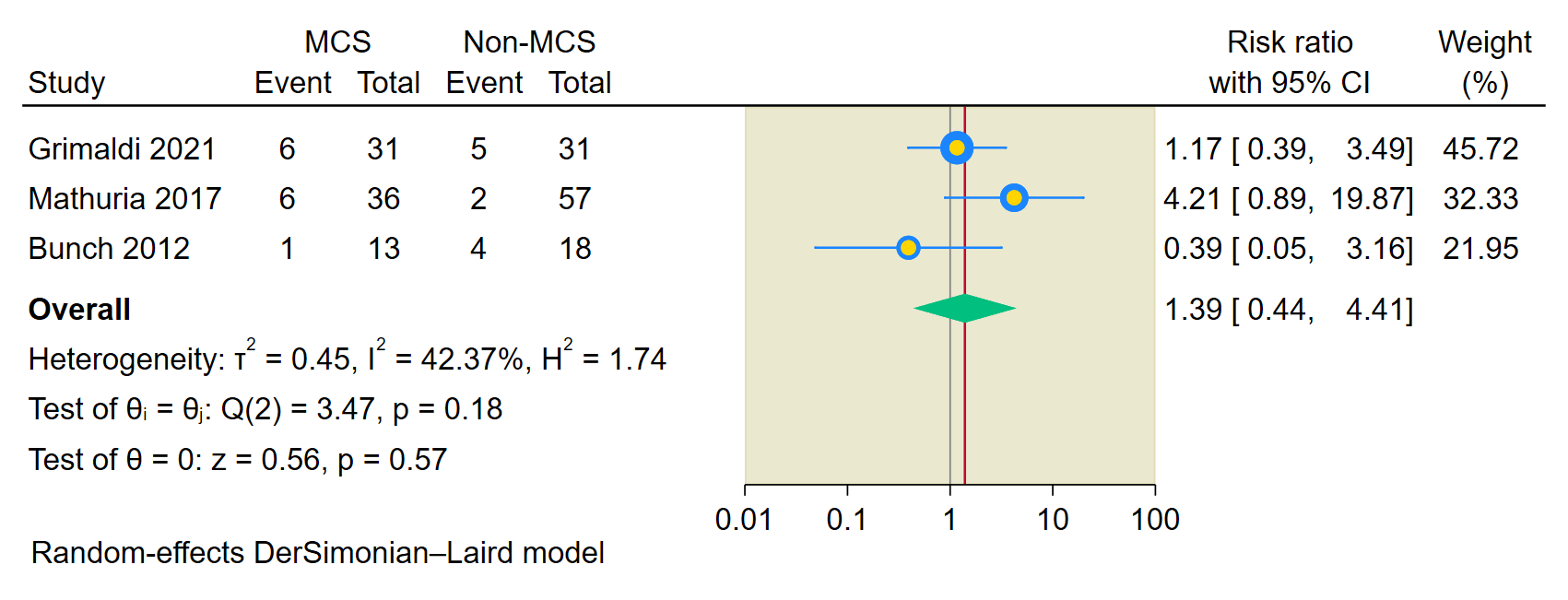


**Figure S12:** Forest plot of orthotopic heart transplant.

Ɵ_1_ refers to assessments of heterogeneity between studies. Ɵ refers to the global test of differences between the treatments.

**
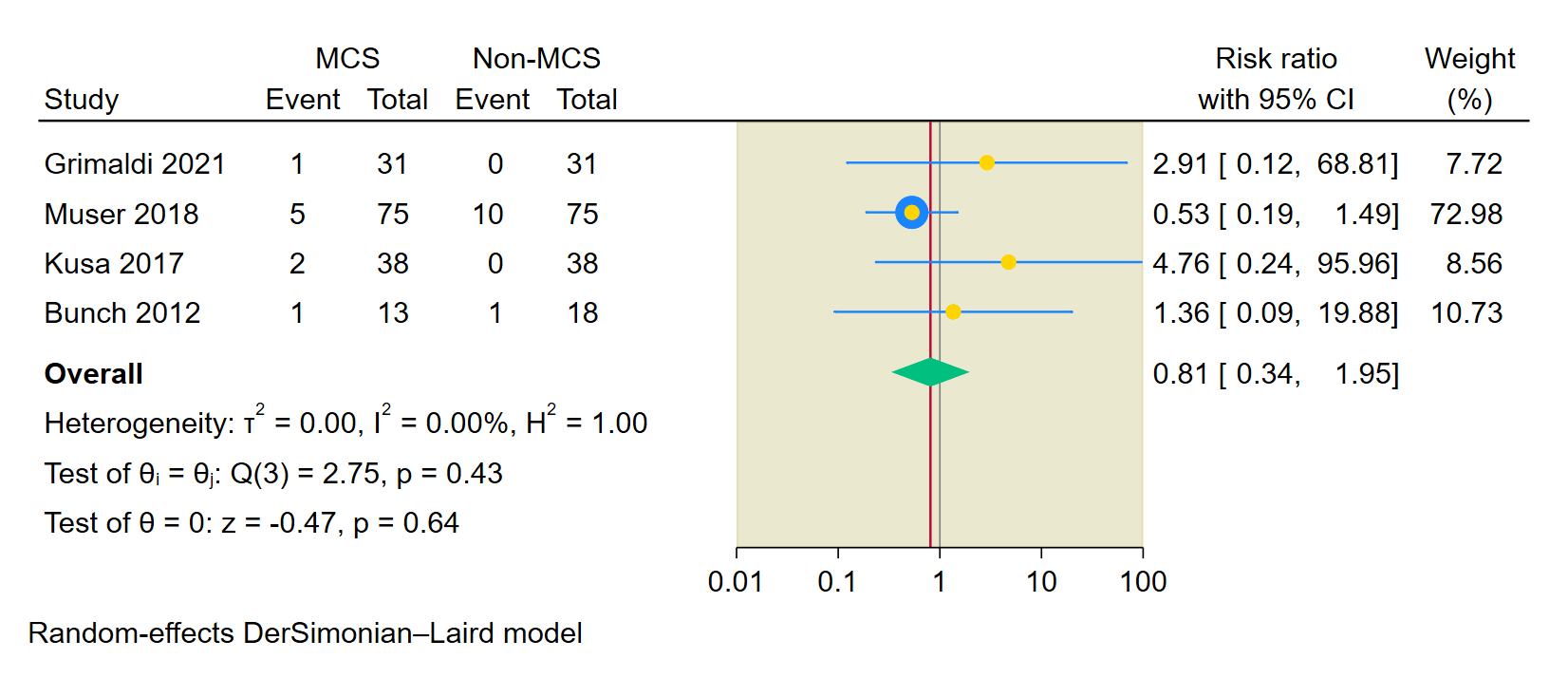
**

**Figure S13:** Forest plot of epicardial ablation.

Ɵ_1_ refers to assessments of heterogeneity between studies. Ɵ refers to the global test of differences between the treatments.

**
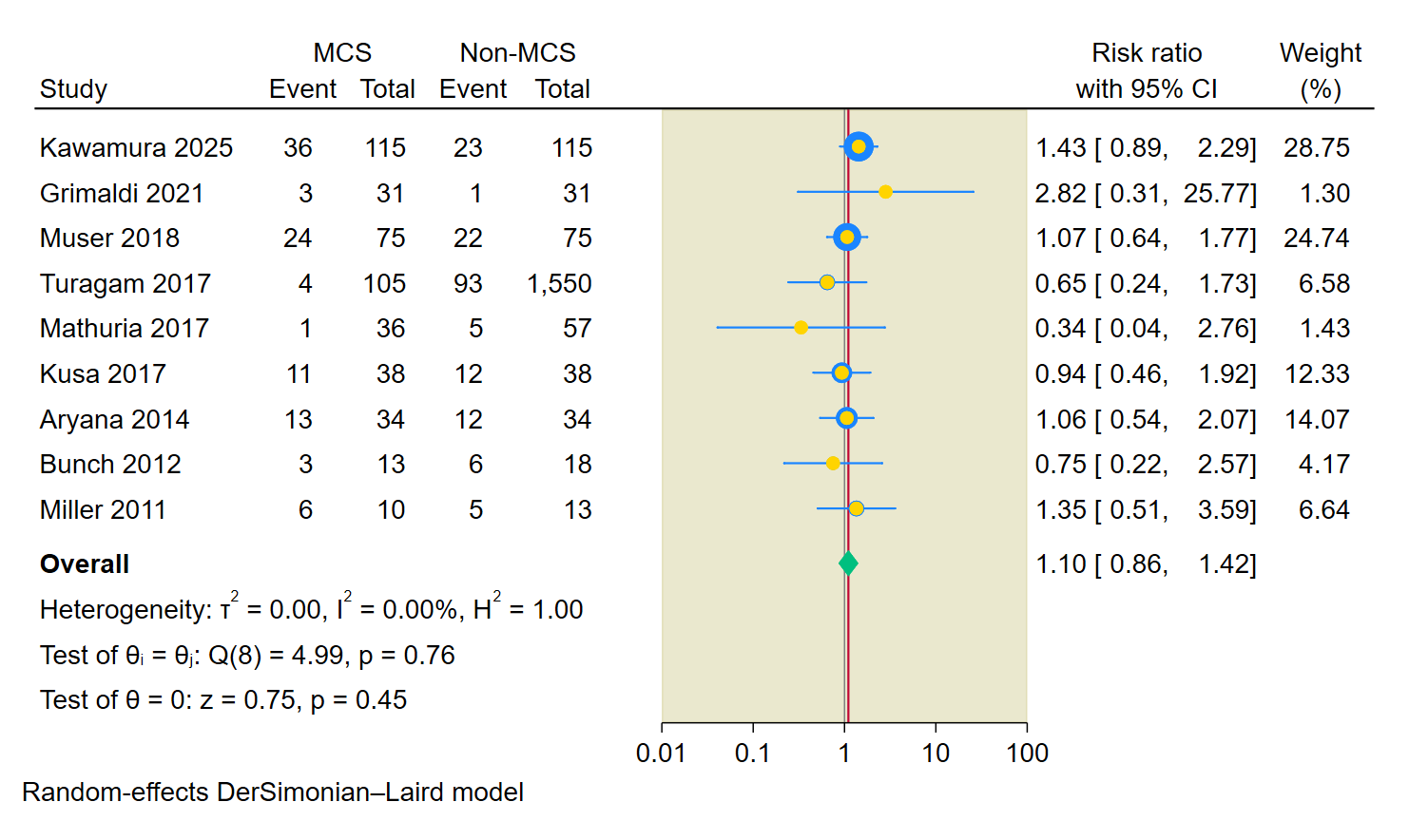
**

**Figure S14:** Forest plot of total fluoroscopy time.

Ɵ_1_ refers to assessments of heterogeneity between studies. Ɵ refers to the global test of differences between the treatments.


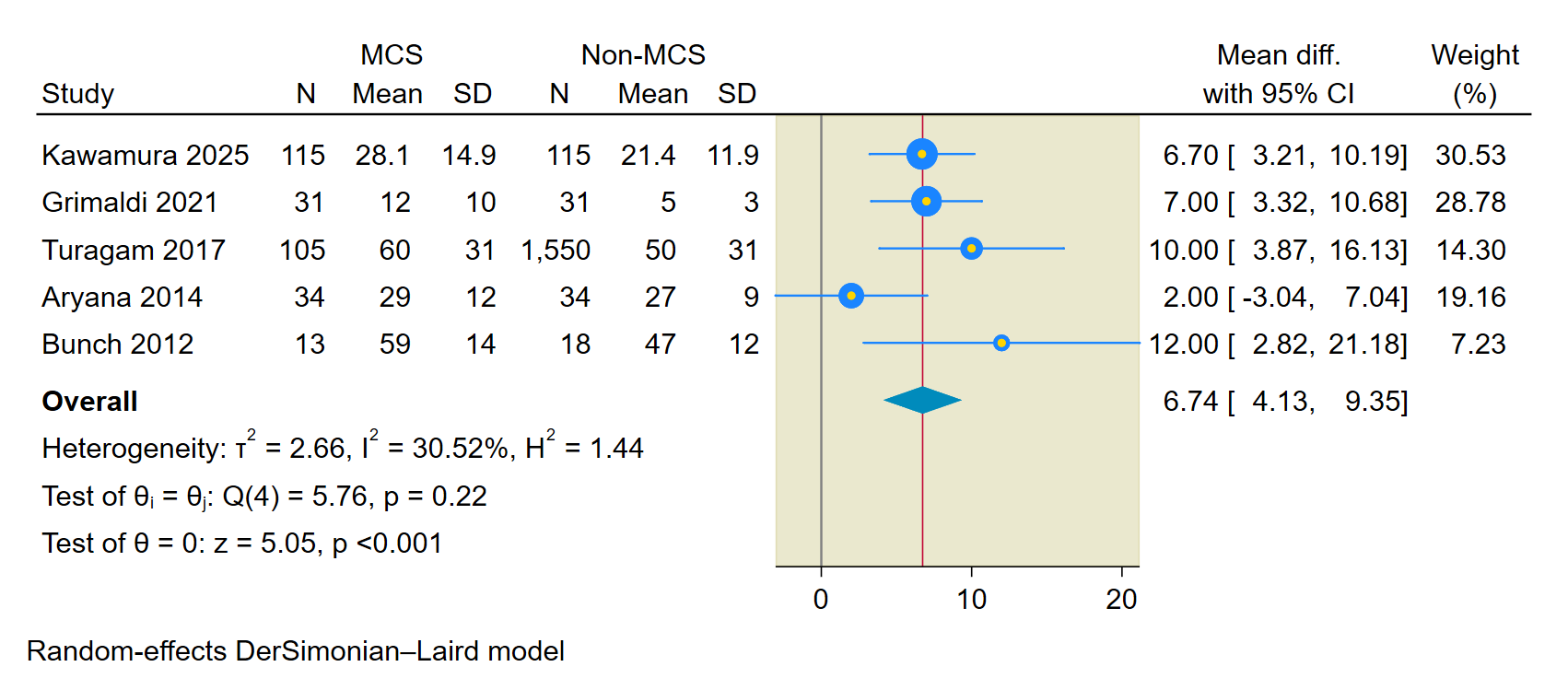


**Figure S15:** Forest plot of total procedural time.

Ɵ_1_ refers to assessments of heterogeneity between studies. Ɵ refers to the global test of differences between the treatments.


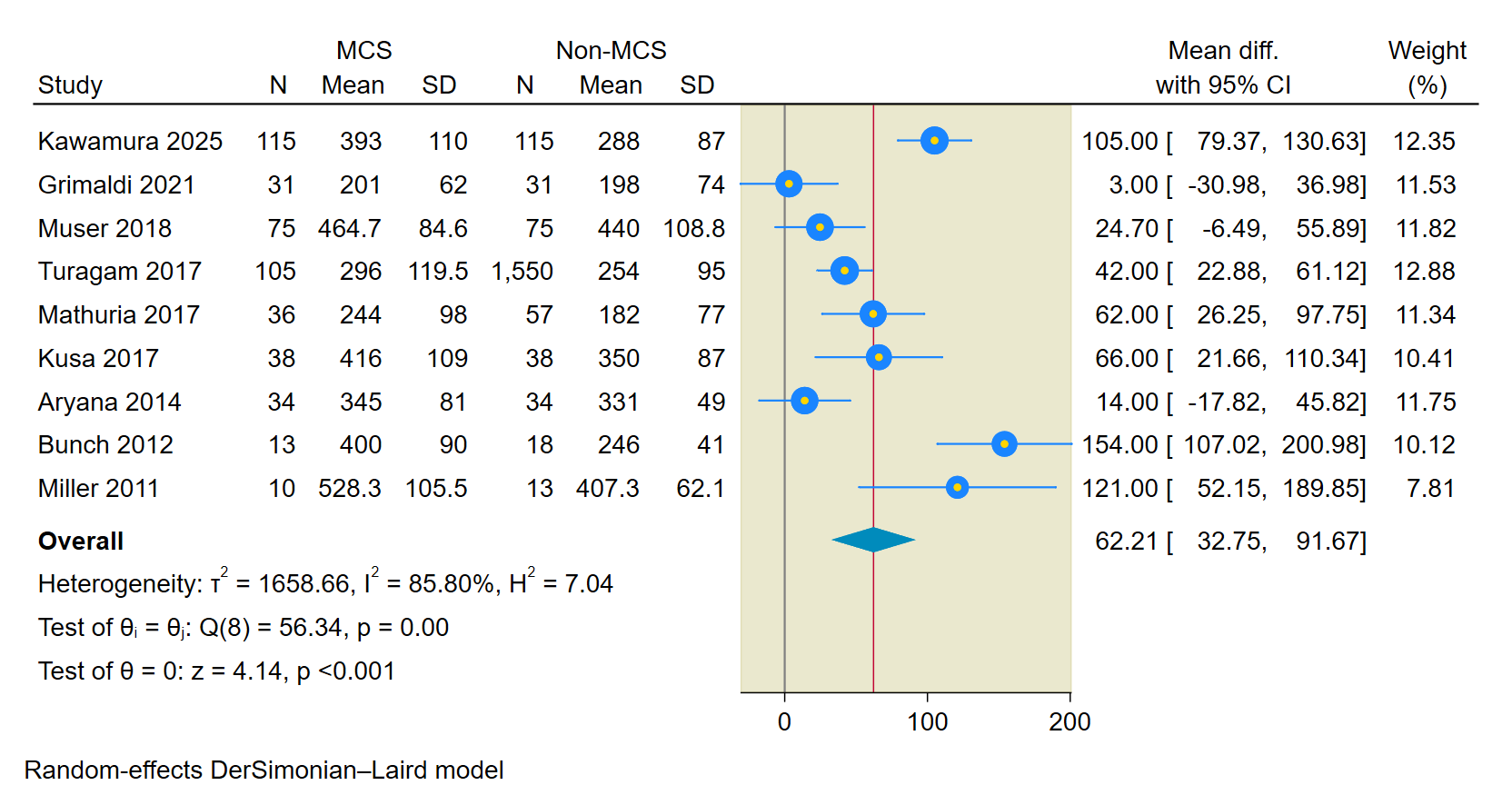


**Figure S16:** Forest plot of VT induction number.

Ɵ_1_ refers to assessments of heterogeneity between studies. Ɵ refers to the global test of differences between the treatments.


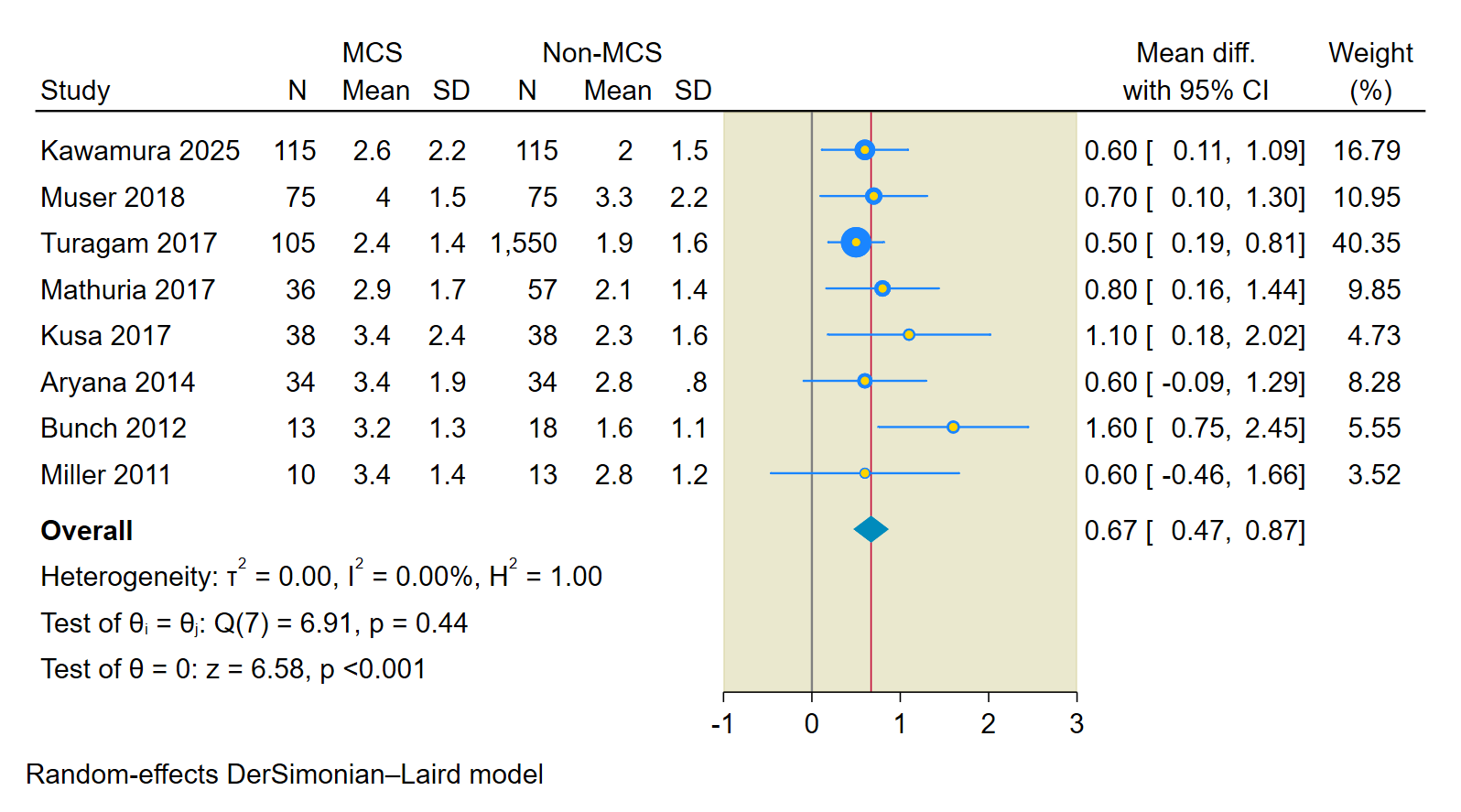


**Figure S17:** Forest plot of VT or VF induction during procedure.

Ɵ_1_ refers to assessments of heterogeneity between studies. Ɵ refers to the global test of differences between the treatments.


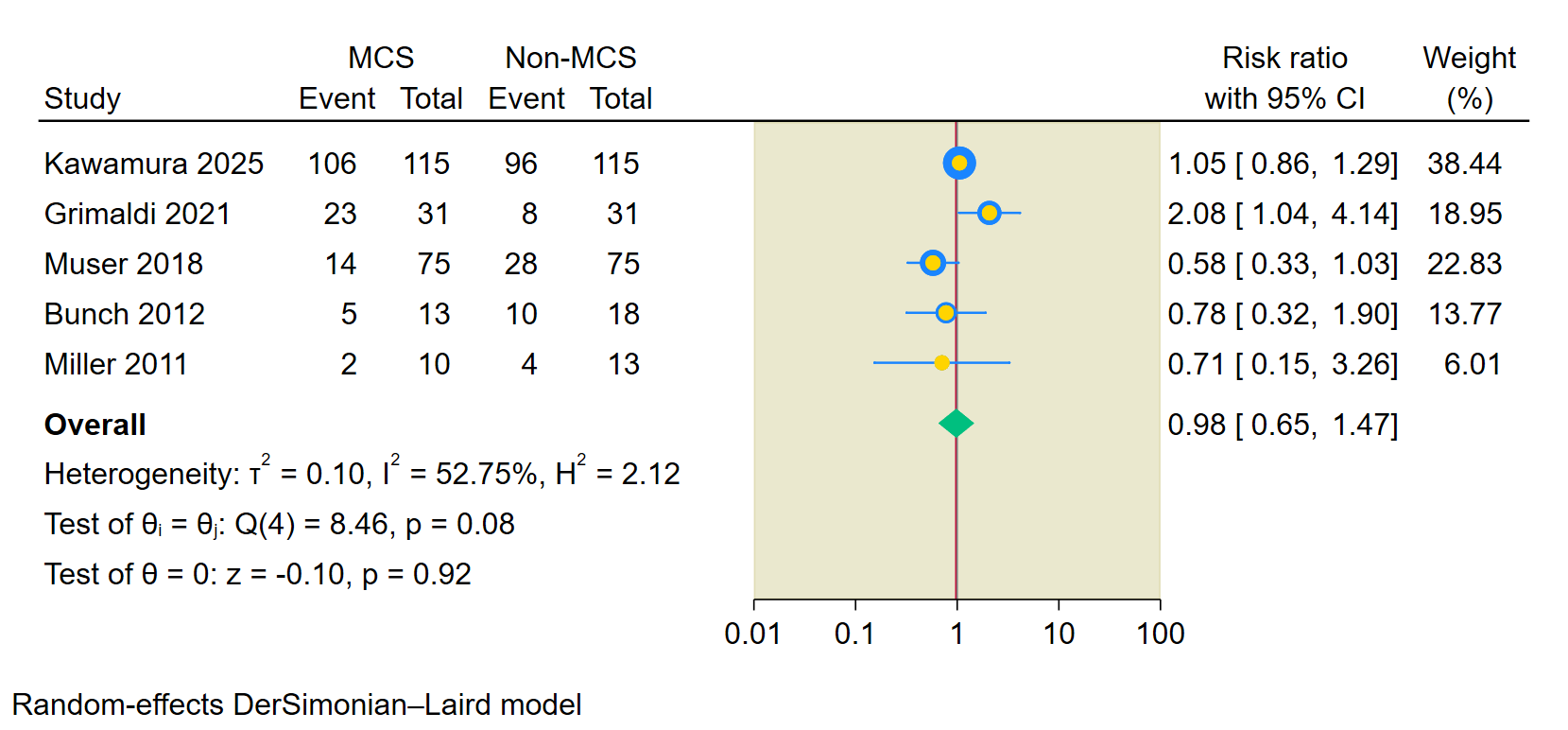
**Figure S18:** Forest plot of VT or VF induction post procedure.

Ɵ_1_ refers to assessments of heterogeneity between studies. Ɵ refers to the global test of differences between the treatments.


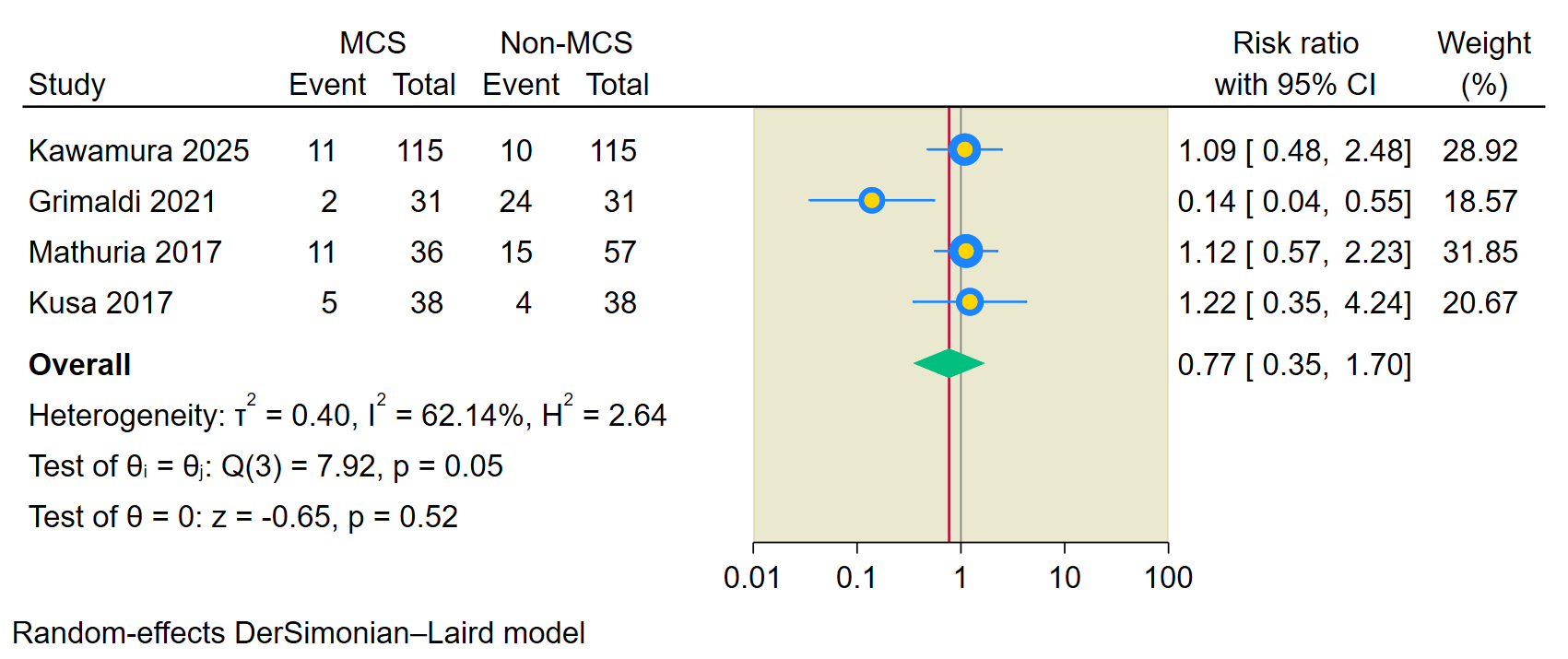


**Figure S19:** Forest plot of Total radiofrequency ablation time.

Ɵ_1_ refers to assessments of heterogeneity between studies. Ɵ refers to the global test of differences between the treatments.


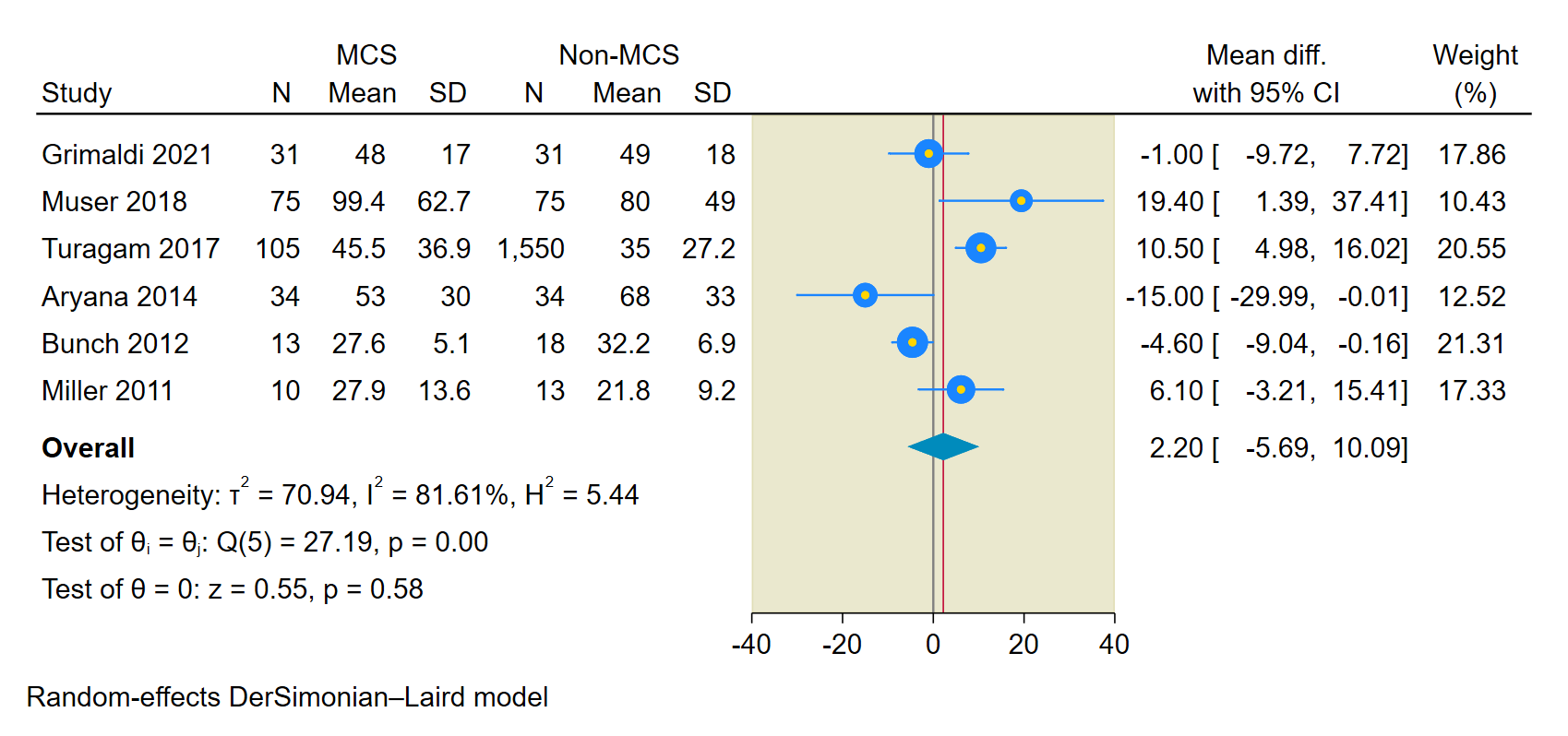

Supplement: Supplementary file 1 — Figure S1: Leave‐one‐out sensitivity analysis of all‐cause death. Figure S2: Funnel plot of all‐cause death. Figure S3: Funnel plot using the trim and fill method for All‐cause death. Figure S4: leave‐one‐out sensitivity analysis of Procedural success. Figure S5: leave‐one‐out sensitivity analysis of VT recurrence. Figure S6: Forest plot of Stroke. Figure S7: Forest plot of Pericardial Effusion or Tamponade. Figure S8: Forest Plot of Periprocedural Complications. Figure S9: Forest Plot of Decompensated Heart Failure. Figure S10: Forest Plot of vascular complications. Figure S11: Forest plot of Cardiovascular Death. Figure S12: Forest plot of orthotopic heart transplant. Figure S13: Forest plot of epicardial ablation. Figure S14: Forest plot of total fluoroscopy time. Figure S15: Forest plot of presence of procedural time. Figure S16: Forest plot of VT induction number. Figure S17: Forest plot of VT or VF induction during procedure. Figure S18: Forest plot of VT or VF induction post procedure. Figure S19: Forest plot of Total radiofrequency ablation time. Table S1: Definition of MACE. Table S2: Procedure Success Definitions. Table S3: Periprocedural Complication Definitions. Table S4: Risk of Bias assessment of observational studies by the Newcastle–Ottawa Scale (NOS). [file JCE-37-1564-s001.docx]
